# MicroRNA-195 inhibits proliferation, invasion and metastasis in breast cancer cells by targeting FASN, HMGCR, ACACA and CYP27B1

**DOI:** 10.1038/srep17454

**Published:** 2015-12-03

**Authors:** Richa Singh, Vikas Yadav, Sachin kumar, Neeru Saini

**Affiliations:** 1 Functional Genomics Unit, CSIR-Institute of Genomics and Integrative Biology (IGIB). Council of Scientific & Industrial Research (CSIR), Delhi, India

## Abstract

De novo lipogenesis, a hallmark for cancers is required for cellular transformation. Further it is believed that resistance to apoptosis and epithelial-to-mesenchymal-transition(EMT) facilitates metastasis via over-expression of anti-apoptotic Bcl-2. Previously we demonstrated that hsa-miR-195 targets BCL2, induces apoptosis and augmented the effect of etoposide in breast cancer cells. However, the mechanism behind its function remains elusive. Herein gene expression profiling was done in presence/absence of hsa-miR-195 in Breast cancer cells. IPA revealed mitochondrial dysfunction, fatty acid metabolism and xenobiotic metabolism signalling among the top processes being affected. For the first time we herein identified ACACA, FASN (the key enzymes of de novo fatty acid synthesis), HMGCR (the key enzyme of de novo cholesterol synthesis) and CYP27B1 as direct targets of hsa-miR-195. We further showed that ectopic expression of hsa-miR-195 in MCF-7 and MDA-MB-231 cells not only altered cellular cholesterol and triglyceride levels significantly but also resulted in reduced proliferation, invasion and migration. We further demonstrated that over expression of hsa-miR-195 decreased the Mesenchymal markers expression and enhanced Epithelial markers. In conclusion we say that hsa-miR-195 targets the genes of de novo lipogenesis, inhibits cell proliferation, migration, and invasion which potentially opens new avenues for the treatment of breast cancer.

Breast cancer is currently the leading cause of cancer death among women worldwide. ER^+^ subtype (Estrogen receptor or hormone receptor subtype) is the most vital discriminator of breast cancer, accounting for nearly 75% of all breast cancer cases^1^. Therapy approaches include irradiation and surgery, with chemotherapy considered an important strategy to treat breast cancer. It is believed that during chemotherapy, drug resistance frequently develops and impairs the successful treatment of breast cancer. In addition there is a need for new therapeutic targets as hormonal therapy is facing challenges against ER^-^ patients as well as acquired drug resistance in ER^+^ patients^2^.

Literature reveals that highly proliferating cancer cells need to synthesize fatty acids de novo for membrane and energy production. De novo fatty-acid synthesis involves two key enzymes, acetyl-CoA carboxylase (ACACA) and fatty-acid synthase (FASN). In normal tissues de novo fatty-acid synthesis is usually suppressed, and FASN expression is maintained at low levels whereas in cancer, the cells are highly dependent on the de novo synthesis^3^. Recently small-molecule BCL-2 inhibitors (ABT-737 etc) and FASN inhibitors such as cerulenin, C75 and orlistat have been shown to induce apoptosis in breast cancer cells both *in vitro* and *in vivo* but heterogeneity of tumor and complexity of signalling pathways remains a major hurdle for effective cancer therapy. Hence there is an urgent need to identify a new generation of anticancer agents^4^,^5^,^6^.

micro RNAs (miRNAs) have emerged as key therapeutic agents against cancers and sufficient evidences are there to show that dysregulation of miRNAs also leads to drug resistance in different cancers and correction of these miRNAs using miRNA mimics or antagomiRs can normalize the gene regulatory network and signaling pathways and sensitize cancerous cells to chemotherapy and may provide exciting opportunities for cancer therapy^7^,^8^.

We and others previously reported that hsa-miR-195 negatively regulates BCL2 expression by binding to its 3′ UTR^9^,^10^. Multiple studies have also shown that hsa-miR-195 regulates biological processes like apoptosis, cell cycle and proliferation by targeting CDK4, CDK6, cyclin D1, cyclin E1, E2F3, E2F5 and WEE1^11^,^12^,^13^. Yang G *et al.* recently showed that expression of miR-195 is low in breast cancer cells (and multidrug-resistant breast cancer tissues) and upregulation of miR-195 increases the sensitivity of breast cancer cells towards chemotherapeutic drug adriamycin^14^.

Results of our study as well as the previous studies suggested the therapeutic potential of this miRNA however despite these studies it remains unclear how the cell’s transcriptome responds to the presence/absence of hsa-miR-195. To understand how hsa-miR-195 exerts regulatory effects we herein, performed gene expression profiling using an Illumina microarray in MCF-7 and -231 cells in presence of hsa-miR-195 or hsa-miR-195 inhibitor (antimiR-195).

In this study, we used two breast cancer cell lines MCF-7 cells (rapidly growing tumor cells that are ER-positive, E-cadherin positive and noninvasive^15^ and MDA-MB-231 (invasive and metastatic tumor cells) that are ER-negative and lacks E-cadherin^16^. Herein, we identified and validated key genes of the de novo lipogenesis as direct targets of hsa-miR-195. Over expression of hsa-miR-195 down regulated and silencing of hsa-miR-195 by antimiR-195 up regulated the expression of ACACA, FASN and 3-Hydroxy-3-Methyl glutaryl CoA reductase (HMGCR), Cytochrome P450 family 27, subfamily B, polypeptide 1 (CYP27B1), a member of the cytochrome P450 superfamily respectively in breast cancer cells. Over-expression of hsa-miR-195 in these cells reduced the cellular cholesterol and triglyceride levels whereas inhibition of endogenous hsa-miR-195 enhanced these metabolites significantly. Our findings provide evidence that microRNA-195 attenuates epithelial-mesenchymal transition (EMT) in breast cancer cells by targeting FASN, HMGCR, ACACA and CYP27B1 and strongly suggest that overexpression of miR-195 may have therapeutic value in treating breast cancer.

## Results

### Transcriptome profiling and Ingenuity Pathway Analysis identifies mitochondrial dysfunction and lipid metabolism as the most significant pathways influenced by hsa-miR-195 in MCF-7 cells

**Figure 1:**
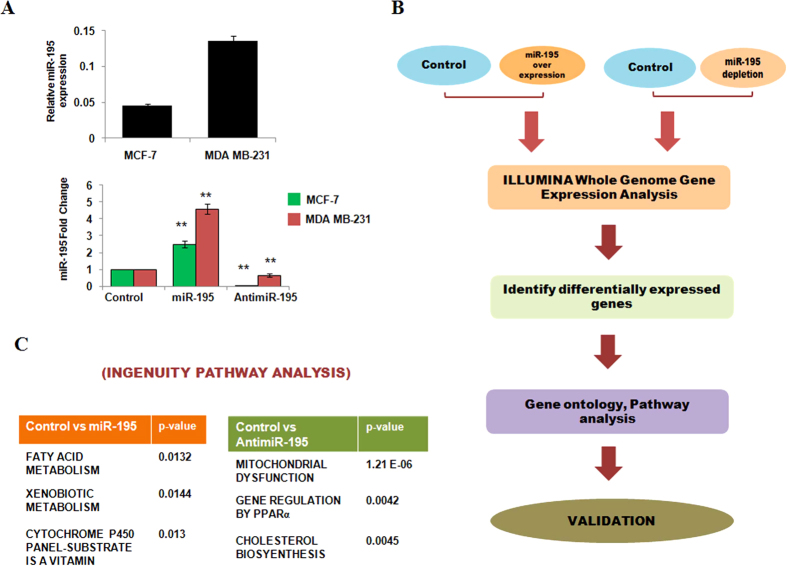
Microarray profiling and transcriptome analysis after altered cellular hsa-miR-195 levels. (**A**) Upper panel represents the endogenous levels and lower panel represents the cellular levels of hsa-miR-195 after overexpression or depletion by transfecting pre-miR-195 clone (p195) or hsa-miR-195 inhibitor, antimiR-195 in MCF-7 and MDA-MB-231 cells as detected by Taqman based quantitative realtime PCR. Graphs are representative of three independent experiments and represent mean fold changes in miR-195 levels with respect to untransfected MCF-7 cells (mean ± S.D). **p < 0.01 (**B**) Outline of study design. (**C**) The top toxicological pathways with their respective log p-values which was predicted by Ingenuity Pathway Analysis tool based on differentially expressed genes after miR-195 overexpression and miR-195 depletion in MCF-7 cells.

Previously our lab has highlighted the therapeutic potential of hsa-miR-195 as an anticancer agent. However, to fully establish its therapeutic function it is necessary to further characterize hsa-miR-195. We herein performed genome-wide gene expression profiling of transfected (miR-195/antimiR-195) and untransfected MCF-7 cells and MDA-MB-231 cells using Illumina HT12 v4 gene expression beadchip. As shown in Fig. 1A, we first checked the endogenous levels as well as the over expression and depletion efficiency of miR-195 in MCF-7 and MDA-MB-231 cells respectively. Consistent to our previous findings^10^, TaqMan based real time PCR assay showed that hsa-miR-195 over expression increases the levels of mature form of hsa-miR-195 by 2.5-fold (*p* value 0.03) and by 4.58-fold (*p* value 0.04) and significant decrease in the mature form of hsa-miR-195 after anti-miR-195 transfection in MCF-7 and MDA-MB-231 cells. We next performed illumina, as shown in schema (Fig. 1B). Analysis of the array data of MCF-7 cells by Illumina Bead Studio software showed 1418 (622 downregulated, mean fold change <0.66; 796 upregulated, mean fold change >1.5) differentially expressed genes (DEG) after over expression of hsa-miR-195 and 428 (DEG) after inhibition of hsa-miR-195 (353 genes were upregulated; mean fold change >1.5:75 genes downregulated; mean fold change <0.66) in MCF-7 cells (Supplementary Figure). The data obtained have been deposited in NCBI’s Gene Expression Omnibus (GEO Series accession number GSE43656). Supplementary Table S2 and S3 show a list of differentially expressed genes that were found in the presence or absence of hsa-miR-195 in MCF-7 cells. To identify functional relationships among the differentially expressed genes, datasets representing genes with altered expression profile derived from microarray analysis were imported into the Ingenuity Pathway Analysis tool (IPA Version 8.8). In IPA differentially expressed genes are mapped to genetic networks available in the ingenuity database and then ranked by score. A network is a graphical representation of the molecular relationships between molecules. Supplementary Table S5 and S6 shows the top associated networks after over expression or inhibition of hsa-miR-195 in MCF-7 cells. Top Tox list tool of IPA was used to identify the pathways and gene lists that were most significant in the dataset. Fisher’s exact test was used to calculate a p-value, determining the probability that each toxicity list assigned to that dataset is not random. Figure 1C (lower panel) shows the top Tox lists obtained from the IPA that were most significant to the dataset of hsa-miR-195 over-expressed and depleted MCF-7 cells respectively. In our analysis, IPA Tox list revealed a significant enrichment of mitochondrial dysfunction, mechanism of gene regulation by peroxisome proliferators via PPAR-alpha, fatty acid metabolism, cholesterol metabolism and xenobiotic metabolism signalling after over-expression or depletion of hsa-miR-195 in MCF-7 cells.

Simultaneously profiling was also done in MDA-MB-231 cells in presence and absence of miR-195 and we found 300 (DEG) after over expression of hsa-miR-195 and 219 (DEG) after inhibition of hsa-miR-195 (Supplementary Figure S2 and Supplementary Table S7-S8). Similar to MCF-7 cells, Tox list of Ingenuity pathway analysis also revealed a significant enrichment of mechanism of gene regulation by peroxisome proliferators via PPAR-alpha and cholesterol biosynthesis (data not shown).

### Over-expression of miR-195 leads to mitochondrial dysfunction

**Figure 2:**
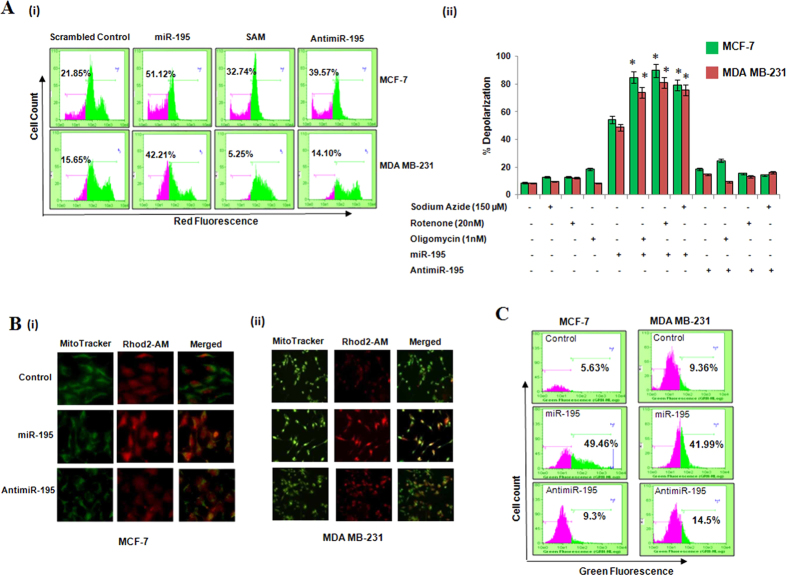
Hsa-miR-195 mediates mitochondrial dysfunction in MCF-7 and MDA-MB-231 cells. (**A**) (i) TMRE staining was carried out to detect mitochondrial membrane depolarization status in hsa-miR-195 overexpressed/depleted breast cancer cells. 24 h post transfection, cells were harvested and stained with 100 nM TMRE as described in Material and Methods. The data is representative of the three independent experiments. (ii) MCF-7 and MDA-MB-231 cells were pre-treated with or without 20 nM rotenone, 150 μM sodium azide or 1 nM oligomycin for 1 h followed by transfection of miR-195 (p195)/anit-miR-195 for 24 h and then the mitochondrial membrane potential was analyzed using TMRE. Bar graph represents mean ± S.D of three independent experiments with *p < 0.01. (**B**) Rhod-2 specific mitochondrial localization of calcium ions after hsa-miR-195 overexpression and inhibition in MCF-7**(i)**, and MDA-MB-231**(ii)** cell lines was determined by fluorescence microscopy as described in Materials and Methods section. (**C**) Fluo3-AM staining of cytosolic Ca^2+^ ions in breast carcinoma cell lines was done as described in Materials and Methods section. The cells were harvested 24 h post transfection and stained with 0.5 μM Fluo-3-AM in HBSS buffer. The data is representative of the three independent experiments with p < 0.05.

Mitochondria is known to play a central role in energy supply for normal cell function and also perform many important cellular physiology tasks such as induction of apoptosis, cellular calcium level and redox homeostasis. Our earlier study and current IPA analysis pointed towards mitochondrial dysfunction in hsa-miR-195 induced apoptosis^10^. To further confirm the role of hsa-miR-195 in regulating mitochondrial dysfunction we first used the indicator TMRE (sensor of mitochondrial inner membrane potential), which is taken up by the mitochondria in a membrane potential dependent manner. Our results demonstrated significant decrease in orange-red fluorescence in hsa-miR-195 over-expressed MCF-7 and MDA-MB-231 cells as compared to scrambled control transfected cells (Fig. 2Ai). Differential sensitivity of miR-195 overexpressing or anti-miR expressing cells towards the mitochondrial function inhibitors like oligomycin, rotenone and sodium azide was also investigated and interestingly electron transport chain (ETC) inhibtiors augmented the effect of miR-195 whereas no change in the mitochodnrial membrane potential was observed when MCF-7 and MDA-MB-231 cells were transfected with anti-miR-195 (Fig. 2Aii).

We further examined the effect of alterations of hsa-miR-195 on calcium levels. Breast cancer cells were dual labelled with mitochondrial calcium specific dye, Rhod-2AM, and mitochondria specific dye MitoTracker Green and as shown in Fig. 2B (Supplementary Figure S2 iii), over-expression of hsa-miR-195 led to significant increase in mitochondrial calcium as compared to control and antimiR-195 transfected MCF-7 and MDA-MB-231 cells. Simultaneously, the cytosolic calcium levels were also measured using Fluo-3AM dye as described in Materials and Methods and we found significant increase in Fluo-3 AM fluorescence after hsa-miR-195 overexpression in MCF-7 and MDA-MB-231 cells, whereas antimiR-195 transfection showed insignificant change in the cytosolic calcium levels as compared to control (Fig. 2C). Taken together all these findings confirmed that over-expression of hsa-miR-195 leads to mitochondrial dysfunction and calcium might play a role in it. Role of calcium in this needs further investigation.

### Hsa-miR-195 plays an important role in regulating lipid homeostasis

**Figure 3:**
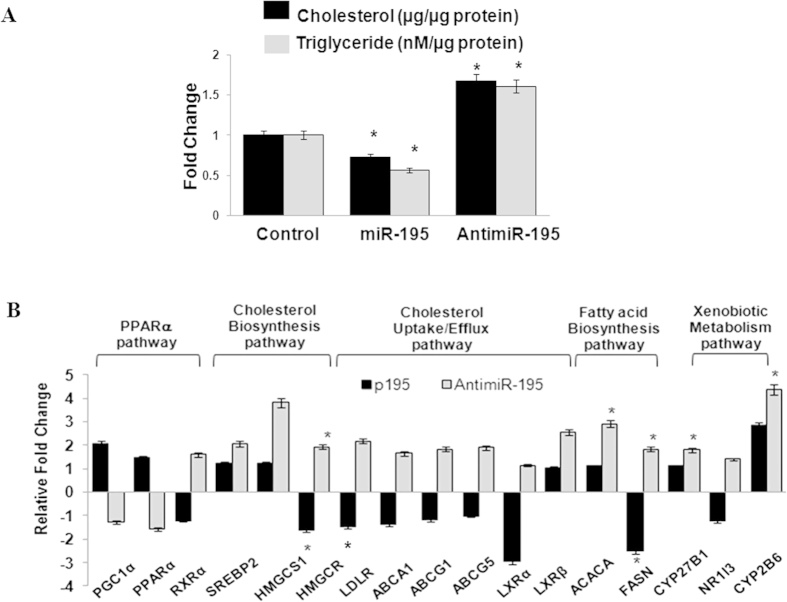
Validation of Microarray profiling and transcriptome analysis after altered cellular hsa-miR-195 levels. (**A**) Cellular cholesterol levels and triglyceride levels were estimated in MCF-7 cells after hsa-miR-195 overexpression and depletion as described in materials and methods section. Relative fold changes as compared to Scrambled miRNA control (in case of miRNA) and scrambled antimiR control (in case of antimiR) are plotted. Bar graphs represent mean ± SD for three independent experiments. *p < 0.05. (**B**) Realtime PCR analysis of selected regulatory genes involved in transcriptional regulation pathways mediated via PPARα, genes involved in cholesterol biosynthesis, uptake and efflux, fatty acid biosynthesis and genes involved in Xenobiotic metabolism via CAR/RXR. 18S rRNA was used for normalization. Graphs represent mean fold changes ± S.D with respect to respective controls i.e. scrambled control for miRNA and scrambled antimiR control for antimiR. *p < 0.05.

To decipher the role of hsa-miR-195 in regulating lipid homeostasis, we first examined the effect of hsa-miR-195 over-expression or inhibition on the cellular levels of cholesterol and triglycerides. We observed statistically significant decrease after overexpression of hsa-miR-195 and increase after antimiR-195 transfection (Fig. 3A) of cholesterol as well as triglyceride levels in MCF-7 cells as compared to scrambled control transfected cells.

We further asked how miR-195 regulated lipid homeostasis (cholesterol as well as triglyceride levels). Several studies suggest that PPARs, liver-X receptors (LXRs) and sterol-response element–binding protein (SREBP) transcription factors regulate lipid homeostasis^17^,^18^,^19^,^20^. Simultaneously there are also reports that SREBPs regulate the expression of genes involved in cholesterol biosynthesis (HMGCS1, HMGCR), cellular uptake (LDLR) and cellular efflux (ATP-binding cassette transporters, ABCA1, ABCG1 and ABCG5)^21^. Hence, we next examined the transcript levels of PPAR-α, PGC1-α (PPAR gamma coactivator 1-alpha), RXR-α, LXR-α, LXR-β, SREBP2, HMGCS1, HMGCR, LDLR, ABCA1, ABCG1, ABCG5, ACACA, FASN and CYP27B1 after over-expression or depletion of hsa-miR-195 in MCF-7 cells using real time PCR analysis (Fig. 3B). Interestingly, we observed significant decrease in the expression of FASN (2 fold, p < 0.05) and HMGCR (p < 0.05) after over-expression of hsa-miR-195. Additionally we observed an increase in the expression of SREBP2, ABCA1, ABCG1, LXR-β and LDLR in MCF-7 cells after anti-miR-195 treatment. There was no significant change in the expression of LXR-α and PPAR-α respectively.

**Figure 4:**
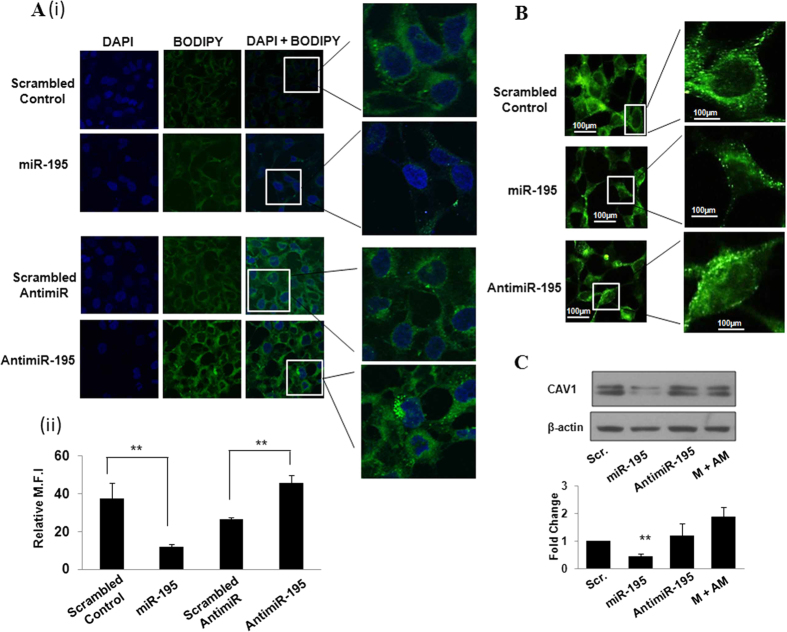
Hsa-miR-195 regulates cellular lipid levels in MCF-7 cells. (**A**) **(i)** BODIPY staining of lipid droplets. Hsa-miR-195 overexpressed and depleted MCF-7 cells were stained with BODIPY and observed under confocal microscope for lipid droplets as described in Materials and Methods section. **(ii)** Fluorescence intensity was calculated by averaging the optical density over at least six region of interests in each sample. Bar graph shows mean fluorescence intensity (M.F.I) ± S.D of BODIPY of three independent experiments. **p < 0.01. here SAM means scramble anti-miR and AM means anti-miR. (**B**) Immunofluorescence assay of CAV1 in MCF-7 cells. Cells were transfected with 195 or antimiR-195 and 24 h post-transfection cells were fixed in 4% formaldehyde solution in PBS for 15 min. Cells were incubated with primary antibody (anti-CAV1 (Abcam, Cambridge, UK) for 2 h followed by Alexa Fluor 488-conjugated secondary antibody (Invitrogen) for 1 h. The fluorescence images were captured using Nikon microscope (Ti Eclipse) at 40x magnification under 488/519 nm excitation and emission wavelengths. Mean fluorescence intensity of CAV1 was calculated by averaging optical densities over six different regions of interest in each sample. Bar graph represents relative mean fluorescence intensity (M.F.I) ± S.D of three independent experiments. **P < 0.01. (**C**) Western blotting for detection of protein levels of CAV1 after overexpression/depletion of hsa-miR-195 in MCF-7 (ER-positive) cells. Bar graph in the lower panel shows mean fold change in CAV1 protein ± S.D of three independent experiments. *P < 0.05.

In the current study cytosolic lipid droplets were also stained with Bodipy and as shown in Fig. 4A, we observed a significant decrease in cytosolic lipid droplets in hsa-miR-195 transfected MCF-7 cells as compared to the cells transfected with scrambled control. On contrary, cytosolic lipid droplets increased substantially when the endogenous levels of hsa-miR-195 were depleted by antimiR-195. The scramble antimiR control did not show any significant changes. These results in addition to validating the analysis confirmed that hsa-miR-195 plays an important role in regulation of lipid homeostasis. In addition we also explored the effect of hsa-miR-195 on Caveolin-1 (CAV1), a scaffolding protein intimately involved with intracellular cholesterol trafficking and whose expression is highly dependent on the availability of cholesterol^21^,^22^. In MCF-7 cells we observed that over expression of hsa-miR-195 led to a 2.3-fold (*P* value = 0.034) decrease in the expression of CAV1 protein (Fig. 4B,C) as compared to scrambled transfected cells and antimiR-195 transfection rescued this effect. Literature reveals that CAV1 can have both cell survival and pro-apoptotic characteristics but the role of CAV1 in hsa-miR-195 induced apoptosis/cholesterol regulation needs further investigation.

### Hsa-miR-195 targets key denovo fatty acid biosynthesis genes, ACACA and FASN

To understand the underlying molecular mechanism behind miR-195 mediated lipid homeostasis, we aimed to identify direct downstream targets of miR-195 using in-silico algorithm. TargetScan 6.2 revealed that key genes of denovo fatty acid biosynthesis, ACACA (ENST00000353139) and FASN (ENST00000306749) have predicted binding sites for hsa-miR-195 in their respective 3′UTRs. ACACA is the crucial rate-limiting enzyme in the fatty acid biosynthesis pathway catalysing the conversion of acetyl-CoA to malonyl-CoA, the first step in fatty acid biosynthesis^23^. FASN is the holozyme generating the basic fatty acid intermediate, palmitate, which is then processed into other longer chained fatty acids. Computational analysis further showed complete complementarity between seed region (2–8 nt) of hsa-miR-195 and the 1231–1237 nt of ACACA 3′UTR and this site and it was found to be conserved across several species (Supplementary Figure S1). Interestingly we also found that FASN has three predicted binding sites of hsa-miR-195 in its 3′UTR and the interspecies homology search showed that the these were conserved across different species of mammals (Supplementary Figure S1).

**Figure 5:**
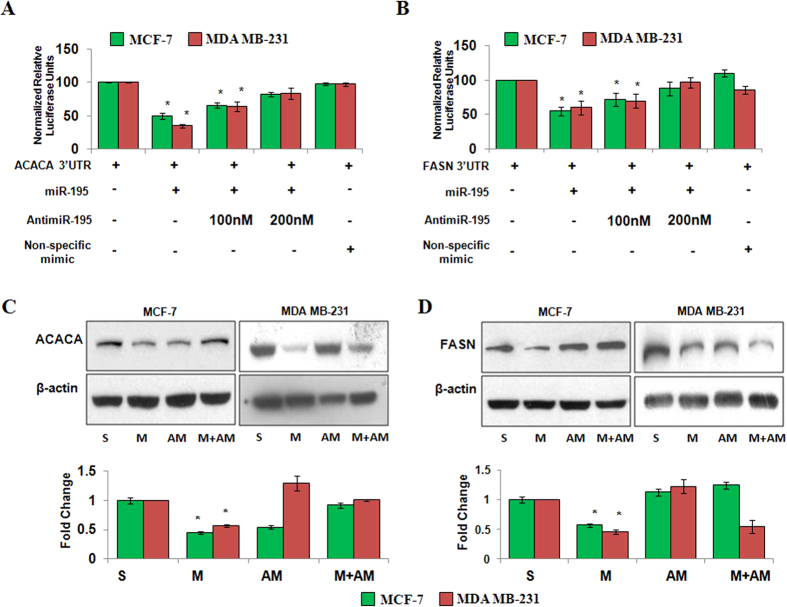
Hsa-miR-195 targets key genes of fatty acid biosynthesis in breast carcinoma cell lines. (**A,B**) Dual luciferase reporter assay of 3′UTR construct of ACACA (**A**), and FASN (**B**), in breast cancer cells. Cells were seeded in 24-well plates and co-transfected with 400 ng of either of luciferase reporter vectors and either 400 ng of pre-miR-195 plasmid alone or in combination with 100 nM antimiR-195, using Lipofectamine 2000 (Invitrogen) as described by the manufacturer. A non-specific mimic control (corresponding to *cel*-67, which has minimal sequence identity with miRNAs in human, mouse and rat) was used as negative control. Relative fold change with respect to UTR itself was plotted. The bar diagram represent mean ± SD for three independent experiments *p < 0.05. **(C**,**D)** Western blotting for detection of protein levels of ACACA (**C**), and FASN(D), after hsa-miR-195 overexpression or depletion in MCF-7 (ER positive) cells and MDA-MB-231 (ER-negative). Bar graphs below the blots represent fold change in protein levels as mean ± SD for three independent experiments. *p < 0.05.

To validate ACACA or FASN as directs targets of miR-195 we obtained reporter constructs with luciferase coding sequence fused to 3′UTR of ACACA/FASN respectively and employed a dual luciferase reporter assay in MCF-7 and −231 cells. Transfection analysis showed that overexpression of p195 led to a 50–65% decrease and antimiR-195 transfection (100 nM) led to 25%–35% increase of lucifease activity of ACACA 3′UTR in these cells (Fig. 5A). A non-specific mimic (cel-miR-67) did not affect luciferase activity of ACACA 3′UTR construct. Similarly Fig. 5B shows that overexpression of p195 reduced the luciferase activity of FASN 3′UTR reporter construct by 45–40% in MCF-7 and MDA-MB-231 cells as compared to only 3′UTR transfected cells and this suppression was relieved by antimiR-195 transfection in both the cells. There was no significant change in luciferase activity when non-specific mimic was transfected.

The effect of miR-195 on protein levels of ACACA and FASN was also tested using western blot analysis. In agreement with the bioinformatic predcitions and luciferase results overexpression of p195 led to 2-fold decrease in protein levels of ACACA and 1.7-fold decrease in protein levels of FASN (Fig. 5C,D) in MCF-7 cells. The protein levels of ACACA and FASN increased when antimiR-195 was cotransfected with p195. Simialr resutls were obtained in MDA-MB-231 cells (Fig. 5C,D). Taken together all these results confirmed that hsa-miR-195 targets key genes of denovo fatty acid biosynthesis, ACACA and FASN.

### HMGCR the rate-limiting enzyme in the biosynthesis of cholesterol is a potential target of Hsa-miR-195

**Figure 6:**
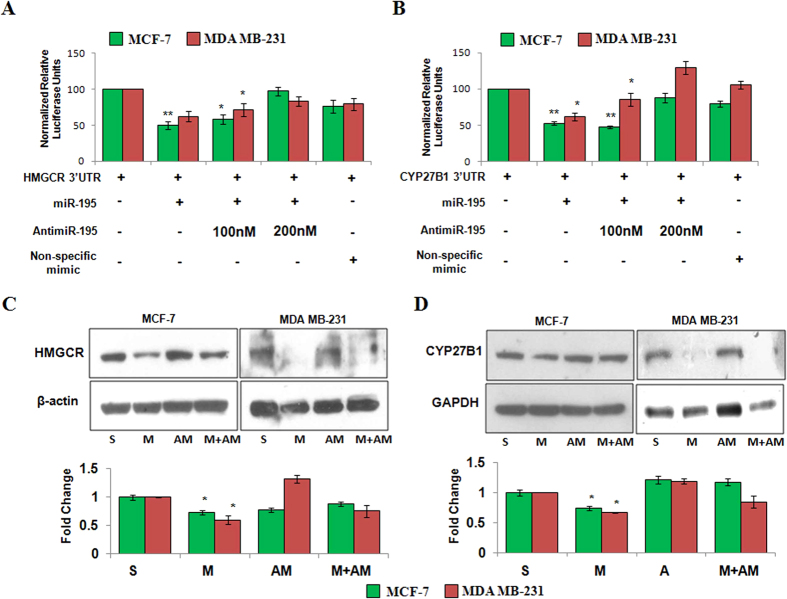
Hsa-miR-195 targets HMGCR and CYP27B1 in breast carcinoma cell lines. (**A,B**) Dual luciferase reporter assay of 3′UTR construct of HMGCR (**A**), and CYP27B1 (**B**), in breast cancer cells. Cells were cotransfected with 3′UTR plasmid and p195 or antimiR-195 or both p195 and antimiR-195. A non-specific mimic was also cotransfected with 3′UTR construct as negative control. Luminscence was measured 24 h post transfection as described in Materials and methods and relative fold change with respect to UTR itself was plotted. The bar diagram represent mean ± S.D for three independent experiments. **p < 0.01, *p < 0.05. **(C,D)** Western blotting for detection of protein levels of HMGCR (**C**), and CYP27B1 (**D**), after hsa-miR-195 overexpression or depletion in MCF-7 (ER positive) cells and MDA-MB-231 (ER-negative). Bar graphs below the blots represent fold change in protein levels as mean ± SD for three independent experiments. *p < 0.05.

Target prediction using miRanda program identified an evolutionarily conserved binding site for hsa-miR-195 in 3′UTR of HMGCR, the enzyme catalyzing rate limiting step of cholesterol biosynthesis^24^. We further observed complete complementarity between the 1388–1394 nt of HMGCR to 2–6 nt of hsa-miR-195 (Supplementary Figure S1). To assess whether HMGCR is a direct target of hsa-miR-195, a dual luciferase reporter assay was performed. As shown in Fig. 6A, overexpression of p195 in MCF-7 cells led to a reduction in luciferase activity by 48% as compared to that of only 3′UTR transfected cells and the antimiR-195 transfection rescued this downregulation in MCF-7 cells. There was no significant change in the luciferase activity when non-specific mimic was transfected. Similar results were obtained in MDA MB-231.

Our western blot analysis demonstrated 1.4-fold decrease in the expression of HMGCR in p195 transfected MCF-7 cells as compared to scrambled control (Fig. 6C). Cotransfection of antimiR-195 and p195 partially rescued this downregulation. Similar results were obtained in MDA-MB-231 (Fig. 6C). Our results confirmed that hsa-miR-195 targets HMGCR.

### Hsa-miR-195 targets CYP27B1

TargetScan algorithm also identified hsa-miR-195 binding site in the 3′UTR of CYP27B1 (ENST00000481739) and 342–348 nt of CYP27B1 3′UTR was found to have perfectly complementary to the 2–8 nt seed region of hsa-miR-195 (Supplementary Figure S1). Dual luciferase reporter assay showed that overexpression of p195 led to a 34% reduction in luciferase activity (Fig. 6B) which was rescued in presence of antimiR-195. Our real time PCR data showed no significant changes in CYP27B1 mRNA levels after hsa-miR-195 overexpression, but inhibition of hsa-miR-195 by antimiR-195 led to a 1.6-fold increase in CYP27B1 levels in MCF-7 cells (Fig. 3B). Furthermore, our western blot data showed 1.37-fold decrease in CYP27B1 protein levels after hsa-miR-195 overexpression in MCF-7 cells (Fig. 6D). Similar results were obtained in MDA-MB-231 (Fig. 6B–D). Our findings suggested that hsa-miR-195 also regulates CYP27B1 by directly binding to its 3′UTR.

### Overexpression of hsa-miR-195 inhibits cell proliferation, migration, invasion and attenuates epithelial to mesenchymal transition in breast cancer cells

**Figure 7:**
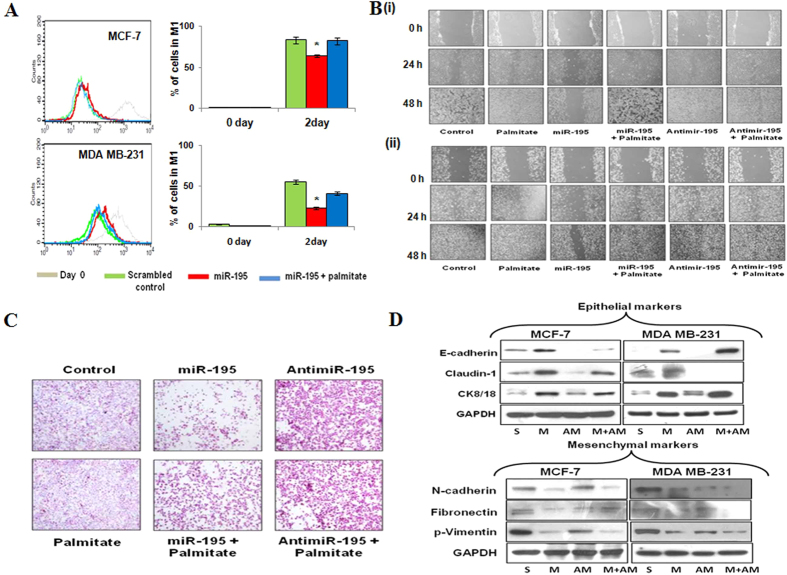
Antiproliferative function of hsa-miR-195 in breast cancer cells. (**A**) MCF-7 and MDA-MB-231 cells were transfected with miR-195 and treated with palmitate (100 μM) for 24 h. CFSE proliferation assay was performed on Days 0 and 2, and proliferation was measured using flow cytometry. The representative overlapped histogram is shown. The data is representative of the three independent experiments with p < 0.05. (**B**) Migration assay in MCF-7 **(i)**, and MDA-MB-231 cells **(ii)** after transfection of miR-195 and antimiR-195 in presence and absence of palmitate (100 μM). The number of cells migrating in the wound increased in untransfected and antimiR-195 transfected cells, whereas fewer cells migrated in the wound area in hsa-miR-195 over-expressed cells. Lower panel shows the graphical representation of quantitative values of the wound size at different time points as determined by the WimScratch module of Wimasis online software. Data shown are representatives of three independent experiments. The error bar represents s.d. from three independent experiments. **p < 0.01, *p < 0.05. (**C**) Invasion Assay was performed as described in materials and method in MDA-MB-231 cells in the presence or absence of miR-195, anti-miR-195 and palmitate (100 μM). (**D**) Western blotting for detection of protein levels of epithelial and mesenchymal markers after hsa-miR-195 overexpression or depletion in MCF-7 (ER positive) and MDA-MB-231 (ER-negative) cells. The data is representative of the three independent experiments.

MCF7 and MDA MB-231 cells transfected with miR-195 showed a significant reduction in cell proliferation at day 2 compared with cells transfected with scrambled control (*P* < 0.05) as measured by CFSE cell proliferation kit. However, MCF7 and MDA-MB-231 cells treated with palmitate had a higher rate of cell proliferation than cells transfected with miR-195 only (Fig. 7A, Supplementary Figure S3).

We next examined the effect of hsa-miR-195 on cell migration by wound healing assay. As expected in the hsa-miR-195 expressing MCF-7 and MDA-MB-231 cells, the rate of cell migration decreased considerably and the filling of the wound area was time dependent, thereby indicating that hsa-miR-195 treatment severely alters the migration ability of the cells (Fig. 7B, Supplementary Figure S4). The presence of palmitate reversed the miR-195 dependent changes in cell migration.

As shown in Fig. 7C the invasion ability of MDA MB-231cells was inhibited after transfection with hsa-miR-195. On the contrary, the invasion ability of MDA MB-231cells increased after treatment with palmitate (Supplementary Figure S5).

Previous studies have revealed that anticancer agents induce mesenchymal to epithelial transition (MET) in cancer cells. After having identified four main downstream targets of miR-195, we further examined which specific phenotype are regulated by miR-195 in breast cancer cells. We employed western blot to detect the expression of epithelial cell markers such as E-cadherin, ck8/18, claudin 1 and mesenchymal cell markers such as fibronectin, N-cadherin and p-vimentin. As shown in Fig. 7D, we found expression levels of fibronectin, p-vimentin and N-cadherin to be significantly decreased in the breast cancer cell lines after hsa-miR-195 overexpression. On contrary, the expression levels of E-cadherin, ck8/18, claudin-1 were increased in the breast cancer cell lines after hsa-miR-195 overexpression.

Together all these results demonstrated that the transient overexpression of hsa-miR-195 inhibited cell proliferation, migration, invasion and attenuates epithelial to mesenchymal transition in MCF-7 (Tumorigenic but non-metastatic) and MDA-MB-231 (Tumorigenic with high metastatic potential). Interestingly, in our array data also we found significant reduction of stemness-related genes such as BMI-1, SOX-2, Twist-1, GLI-2, CXCR7 and ALDH1A3 after miR-195 overexpression thereby strongly implicating role of hsa- miR-195 in inhibiting breast cancer stem cell-like self-renewal

However this molecular aspect of miR-195 needs further validation.

## Discussion

Despite substantial progress in understanding the breast cancer signaling network, effective therapies remain scarce due to tumor heterogeneity, insufficient disruption of oncogenic pathways, drug resistance and drug-induced toxicity. Hence new and additional approaches are required for the treatment of breast cancer.

High proliferation rate has been the characteristic feature of cancer cells which renders their need for high levels of fatty acid as a substrate for energy production and membrane biogenesis^25^. Cancer cells prefer to derive their need for fatty acid via de novo lipogenesis in comparison to normal human tissues which prefers to utilize exogenous lipids, as a consequence of which cancer cells harbor high levels of FASN and ACACA. Comparatively among various cancer sub types, breast cancer cells significantly nurture very high levels of these key enzymes owing to their high proliferation, aggressiveness and metastatic potential^26^.

We and others have previously reported that hsa-miR-195 negatively regulates BCL2 expression and we also demonstrated that over-expression of hsa-miR-195 not only caused an increase in apoptosis but also augmented the death inducing effect of etoposide in breast cancer MCF-7 cells^10^. In the current study we show that hsa-miR-195 targets key enzymes of the de novo lipogenesis pathways e.g., ACACA, FASN, HMGCR and CYP27B1 by binding to their respective 3′UTRs.

ACACA and FASN are highly expressed in cancers and small molecules targeting these enzymes are being considered as potential therapeutic agents for therapy^27^,^28^,^29^. FASN a multifunctional enzyme plays a key role in biosynthesis of fatty acid and ACACA is known to specifically interact with the protein coded by one of the major breast cancer susceptibility genes BRCA1^30^. Furthermore, FASN is highly elevated in 30% of HER2 over-expressing breast cancers and considerable interest has developed in searching for novel FASN inhibitors as therapeutic agents for treatment of HER2-overexpressing breast also^31^. In the current study we observed significant down regulation of ACACA and FASN after over-expression of hsa-miR-195 in MCF-7 and MDA-MB-231 cells. Recently Mao *et al.* have shown that miR-195 also inhibits cell invasion and migration by down-regulating FASN in osteosarcoma cells^32^. Similar to Mao *et al.* in the current study we also observed that over expression of hsa-miR-195 in breast cancer cells inhibited cell migration, invasion and cell proliferation. We also observed that transient overexpression of hsa-miR-195 inhibited cell invasion in MDA-MB-231 cells. Simultaneously our work also identifies hsa-miR-195 as a regulator of cholesterol metabolism. We observed that hsa-miR-195 targets the rate-limiting enzyme of the mevalonate pathway, HMGCR, by binding to its 3′UTR and down regulating its protein levels. Our experimental findings showed that antimiR-195 treatment not only leads to increase in HMGCR and HMGCS1 mRNA levels but also leads to significant increase in the cholesterol levels. Several recent studies show that aberrant regulation of cholesterol homeostasis has been associated with multiple types of cancer and literature also reveals that statins originally developed as cholesterol lowering drugs that target HMGCR are currently being investigated as potential anticancer agents including breast cancer^33^,^34^.

Our study also identified CYP27B1 to be a novel target of hsa-miR-195. CYP27B1 is a cytochrome P450-containing hydroxylase expressed in kidney and other tissues that generates active vitamin-D [1α, 25(OH)_2_D_3_] from an inactive vitamin-D precursor 25-hydroxycholecalciferol [25(OH)D_3_] and plays an important role in calcium homeostasis, steroid biosynthesis, xenobiotic metabolism etc^35^. Interestingly recently Lopes N *et al.* observed that CYP27B1 is upregulated in breast tumours as compared with normal tissue^36^. In our study we not only observed significant down regulation of CYP27B1 at the transcriptional as well as translational levels but also significant increase in the mitochondrial calcium levels in MCF-7 and MDA-MB-231 cells after over-expression of hsa-miR-195. Furthermore Ingenuity Pathway Analysis of our microarray data (GEO accession number GSE43656) showed CAR/RXR activation after antimiR-195 treatment and we show here that antimiR-195 treatment led to an increase in the expression of constitutive androstane receptor (NR1l3, CAR) and its target gene CYP2B6 at the transcriptional levels in MCF-7 and MDA-MB-231 cells. Since, CAR, a xenobiotic-activated transcription factor is found to be highly expressed in ERalpha-positive breast tumors; future studies in this direction may provide conclusive answers.

**Figure 8:**
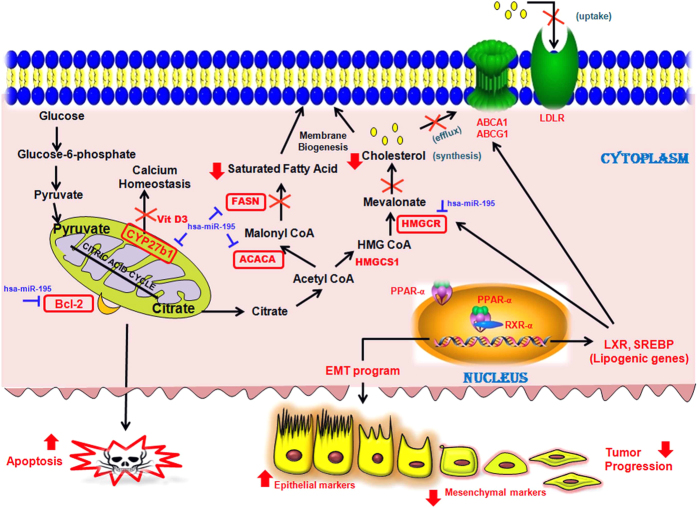
Schematic representation showing various regulatory functions of hsa-miR-195. Model showing different regulatory functions of hsa-miR-195. As per the model when hsa-miR-195 levels rise, the cholesterol and fatty acid biosynthesis processes decrease due to targeting of key regulatory genes of both of these pathways. Our previous study had shown BCL2, an apoptosis regulator, to be a target of hsa-miR-195. Additionally we observed that alterations in hsa-miR-195 levels also lead to changes in expression levels of genes involved in xenobiotic metabolism, vitamin-D metabolism and EMT (epithelial to mesenchymal transition). These functions are represented by bold lines in the model. Genes validated by Real Time PCR are marked in red colour, whereas genes validated by RT-PCR, western blotting and luciferase assay are highlighted by red colour boundary wall.

In conclusion, we hereby present a model (Fig. 8) which reveals that over-expression of hsa-miR-195 leads to a decrease in cholesterol and fatty acid biosynthesis and vice versa by targeting key regulatory genes of both of these pathways. We also speculate that changes in the hsa-miR-195 levels might also change the expression of the genes involved in xenobiotic and vitamin D metabolism. Further studies in this direction may provide a conclusive answer.

The role of pro-apoptotic hsa-miR-195 in inhibiting de novo lipogenesis via targeting genes overexpressed in breast cancer (BCL-2, FASN, ACACA, HMGCR, CYP27B1) makes hsa-miR-195 an effective anticancer molecule. The therapeutic potential of hsa-miR-195 for breast cancer is further strengthened by our migration/wound healing, cell proliferation assay and EMT immunoblot assay. The results in current study provide a detailed insight into functional role of hsa-miR-195 and we believe that hsa-miR-195 can be used as therapeutics for breast cancer. Still these findings remain open for investigation and future *in vivo* studies in this direction may provide conclusive answers. In addition our characterization of hsa-miR-195 enhances our understanding of intricate regulatory networks of cholesterol and fatty acid biosynthesis also. Although our findings point to the basic and translational relevance of miRNA, therapeutic *in vivo* manipulations of hsa-miR-195 are warranted.

## Methods

### Cell culture and transfections

Human Estrogen Receptor (ER)-positive MCF-7, (ER)-negative MDA-MB-231 breast cancer cells were procured from National Centre for Cell Sciences (NCCS), Pune, India and passaged in the lab. The cell lines were not reauthenticated by the authors. Cells were maintained in DMEM containing 10% (v/v) fetal calf serum, 100 Units/ml penicillin, 100 μg/ml streptomycin, 0.25 μg/ml amphotericin at 37 °C in a humidified atmosphere at 5% CO_2_. Transfections were done using Lipofectamine 2000/Lipofectamine LTX-Plus (Invitrogen, CA, USA) reagent according to manufacturer’s protocol.

### Over expression and knock down of hsa-miR-195

For over-expression of hsa-miR-195, a 237 bp region harbouring pre-miR-195 was cloned in pSilencer 4.1 vector (Ambion, Austin, TX, USA) and called p195 throughout the manuscript. Scramble control is a pSilencer 4.1 plasmid containing a scrambled sequence (which has limited homology to known human or mouse sequences) was used as negative control in the experiments. For experiments with antimiR-195, anti-miR^TM^ miRNA inhibitor of miR-195 was purchased from Ambion (Austin, TX). An antimiR miRNA inhibitor negative control (AM17010) (Ambion, Austin, Texas) having a random sequence validated to produce no identifiable effects on known functions of miRNAs was used and was called SAM. 24 h post transfection cells were harvested by trypsinization and used.

### Real-time PCR

Total RNA was extracted with the Trizol reagent (Invitrogen, USA) and reverse transcribed using M-MuLV reverse transcriptase as per the manufacturer’s instructions (Fermentas, USA). TaqMan microRNA assays (Applied Biosystems, CA, USA) that include specific RT primers and TaqMan probes were used to quantify the expression of mature miR-195 (AB Assay ID 000494), as described by the manufacturer. 18S rRNA (AB Assay ID 4333760F) was used for normalization. Primers used for detection of expression levels of ACACA, FASN, HMGCR, CYP27B1, HMGCS1, SREBP1, SREBP2, PGC1-α, PPAR-α, LXR-α, RXR-α, IDI1, SQLE, LDLR, ABCA1, ABCG1, ABCG5, NR1L3 (CAR), CYP2B6 and 18S rRNA are listed in Supplementary Table S1. The real-time PCR data was analyzed using Pfaffl’s method^37^.

### Illumina microarray and Ingenuity pathway analysis

Genome-wide expression profiling was performed using Illumina HumanHT-12 v4 BeadChips (Illumina, CA, USA) representing 47231 human transcripts in three biological replicates of untransfected, miR-195 over expressed and miR-195 depleted (100 nM antimiR-195) MCF-7 and MDA-MB-231 cells as described previously^38^. Data was normalized by rank invariant method and the genes which crossed the threshold of detection p value ≤ 0.05 among all the samples and differential score p value ≤ 0.05 among the test samples were considered to be differentially expressed genes and selected for further investigation. The data obtained has been deposited in NCBI’s Gene Expression Omnibus and is accessible through the Gene Expression Omnibus Series accession number GSE43656. Datasets representing genes with altered expression profile derived from microarray analyses were imported into the Ingenuity Pathway Analysis Tool (IPA Tool; Ingenuity®Systems, Redwood City, CA USA; http://www.ingenuity.com) and analysed as described previously^39^.

### Tetramethylrhodamine ethyl ester (TMRE) staining for mitochondrial membrane potential

Mitochondrial membrane potential was measured using TMRE (Molecular Probes, OR, USA) according to the manufacturers’ protocol. TMRE is a cell permeant, positively charged, red-orange dye that readily accumulates in active mitochondria due to their relative negative charge. Depolarized or inactive mitochondria have decreased membrane potential and fail to sequester TMRE. Consequently, mitochondrial depolarization is indicated by a decrease in the red fluorescence intensity. Briefly, transfected or untransfected cells were stained with TMRE dye at a final concentration of 100 nM for 20 min in dark at 37 °C and then harvested by trypsinization. Cells were washed with 1× PBS and then the red fluorescence was measured by flow cytometry and analysed as done previously (Guava Easycyte, Millipore)^40^. A total of 5,000 events were counted. Wherever indicated Electron transport chain (ETC) inhibitors, such as rotenone (a complex I inhibitor), sodium azide (a complex IV inhibitor), and oligomycin (a complex V inhibitor) were also used.

### Cytosolic and mitochondrial calcium estmation

The change in calcium ion concentration in the cytosol was determined using Fluo-3 AM (Santa Cruz Biotechnology, CA USA). Fluo-3 acetoxymethyl (AM) ester is a cell permeable analog of Fluo-3. After crossing the membrane, the product is quickly metabolized by cytoplasmic esterases to the membrane impermeant Fluo-3 a fluorescence chelator which when excited by visible light (~488 nm) emits a green fluorescence (~525 nm) upon binding with calcium ions. It does not emit fluorescence unless bound to calcium ions. The cells grown in 12-well plates were washed three times with ice-cold HEPES-buffered Hank’s balanced salt solution (HBSS) and were loaded with 0.5 μM Fluo-3 AM in HBSS for 20 min at 37 °C in the dark. After the incubation, twice the volume of HBSS was added to it and cells were further incubated for 40 min. The cells were then washed with HBSS and kept at 37 °C for 10 min. Following this, the cells were trypsinized and fluorescence was detected using Flow cytometer (Guava Technologies, Hayward, CA USA). A minimum of 5,000 events were counted^38^.

To detect mitochondrial calcium levels, Rhod-2 AM (Invitrogen, CA USA) dissolved in DMSO was used. MCF-7 and MDA-MB-231 cells were incubated with fresh medium containing 2 μM of Rhod-2 AM for 30 min in dark and then after two washings of 10 min each a further counterstaining with 200 nM MitoTracker Green (Invitrogen, CA USA) was done at 37 °C for 10 min. Fluorescence images were obtained by fluorescent microscopy (Nikon Inc., NY USA) at 60× magnification under corresponding excitation and emission wave lengths.

### BODIPY staining of lipid droplets

Cytosolic lipid droplets were stained using BODIPY 493/503 (4,4-Difluoro-1,3,5,7,8-Pentamethyl-4-Bora-3a,4a-Diaza-*s*-Indacene) (Molecular Probes, Invitrogen, CA USA) according to the manufacturers’ protocol. Cells were then mounted using Prolong Gold Anti-fade reagent (Invitrogen) and observed under 63× in Carl Zeiss LSM510 Meta confocal microscope. Images were acquired using Zen2000 software.

### Cellular cholesterol and triglycerides levels quantitation

Cholesterol and triglyceride content was determined using cholesterol quantitation kit (Biovision, CA, USA) and Triglyceride quantification kit (BioVision) as mentioned earlier^41^. In brief, 10^6^ cells were lysed and lipids were extracted by homogenization with 200 μl of chloroform: isopropanol: Triton X-100 (7:11:0.1). These lipid extracts were vacuum dried for 30 min and the residues were dissolved in 200 μl cholesterol Reaction Buffer provided with the kit. Cholesterol was estimated by spectrophotometry at λ = 570 nm in a 96 well plate according to the manufacturer’s instructions. For triglycerides levels quantitation, ~10 million cells were lysed in 1 ml solution containing 5% NP-40 in water and slowly heated to 80–100 °C. This extract was then used for fluorometric estimation at Ex/Em = 535/590 nm. Cholesterol and triglycerides content were normalized by total protein concentration.

### Immunofluorescence analysis

Cells were seeded and grown on 25 × 25-mm cover slips in 6-well tissue culture plates. Next day, cells were transfected with p195 or antimiR-195 and 24 h post-transfection, the cells were fixed in 4% formaldehyde solution in PBS for 15 min. Cells were permeabilized for 5 min in 0.1% Triton X-100 followed by 3 washes with 1× PBS. Cells were incubated in blocking buffer (1% BSA and 0.1% Triton X-100 in 1× PBS) for 1 h, primary antibody anti-CAV1 (Abcam, Cambridge, UK) for 2 h followed by Alexa Fluor 488-conjugated secondary antibody (Invitrogen) for 1 h. The fluorescence images were captured using inverted microscope Nikon ECLIPSE Ti (Nikon Corporation, Tokyo, Japan at 40× magnification under 488/519 nm excitation and emission wavelengths.

### Luciferase reporter assay

3′UTR reporter clones for ACACA, FASN, HMGCR and CYP27B1 were obtained from Origene (SC218496, SC210244, SC216360 and SC210195 respectively) and luciferase activity was measured using dual luciferase reporter assay system (Promega, Madison, WI, USA) as described previously^42^.

### Protein preparation and Western blotting

Cells were trypsinized and lysed with modified RIPA buffer (50 mM Tris–HCl, pH 7.4, 150 mM NaCl, 1% NP40, 0.25% Na-deoxycholate, 1 mM EDTA) containing protease inhibitors (1 μg/ml aprotinin, 1 μg/ml leupeptin, 1 μg/ml pepstatin, 1 mM PMSF, 1 mM sodium orthovanadate and 1 mM sodium fluoride) for 30 minutes on ice. The lysates were centrifuged at 16000 × *g* for 30 minutes at 4 °C and the supernatant was collected. Protein concentration was determined by BCA (Sigma, USA) method. Equal amount of proteins (30–50 μg) were separated on 12–15% sodium dodecyl sulphate polyacrylamide gel electrophoresis (SDS-PAGE) and transferred to PVDF membrane (Mdi; Advanced Microdevices, India). Membrane was blocked using 5% skimmed milk for 1 h at RT and incubated with respective antibodies in 1% skimmed milk for 2–3 h followed by incubation with proper secondary antibody for 1 h. Primary antibody for FASN, Fibronectin, E-cadherin, N-cadherin were obtained from Santa Cruz (Santa Cruz Biotechnology, CA, USA). ACACA, CYP27B1, HMGCR, CAV1 were from Abcam (Cambridge, UK), claudin-1, CK8/18, p-Vimentin were from Cell signalling Tech (USA). β-actin and GAPDH were from Sigma (Sigma, USA). β-actin/GAPDH was used as normalizing controls. The primary antibody for β-actin was purchased from Sigma (Sigma, USA). The secondary antibodies were HRP-linked and blots were developed using enhanced chemiluminiscence (Pierce, Amersham). Integrated density values were calculated using AlphaImager 3400 (Alpha InnoTech, San Leandro, CA, USA). These values were then normalized to β-actin. All experiments were repeated at least three times; representative results are presented^43^.

### Palmitate-BSA Conjugate and cell treatment

Sodium palmitate (Sigma, MO, USA) was dissolved in 0.1 M NaOH by heating at 70 °C for 20 min. Solution was then diluted with 10% fatty acid free-BSA (dissolved in autoclaved MilliQ water) and allowed to conjugate for 15 min at 55 °C. Palmitate-BSA conjugate solution was filter sterilized and stored at −20 °C for future use but were allowed to activate at 55 °C for 10 min prior to use in cell culture. In all the experiments palmitate was used at a concentration of 100 μM.

### Cell proliferation assay

To detect proliferation rate, MCF7 and MDA-MB-231 cells were transfected with p195 and in presence or absence of palmitate cells were labelled with 5 μM Carboxyfluorescein Diacetate (CFSE) dye (CellTrace™ CFSE Cell Proliferation Kit, C34554, Invitrogen) for 10 min at 37 °C and the staining was ended by adding 5 volumes of ice-cold culture media to the cells. CFSE proliferation assay was performed on Days 0 and 2, and proliferation was measured using flow cytometry. The representative overlapped histogram is shown.

### Wound healing assay

MCF7 and MDA-MB-231 cells were plated on a six-well tissue culture plate. The next day, cells were transfected with p195 or antimiR-195. After 24 h, the wound was artificially created by scraping with a sterilized pipette tip and the fresh media (in presence or absence of 100 μm palmitate) was added to the cells after washing with PBS respectively. The wound area was monitored for the next 2 days at an interval of 24 h. The microscopy images of the cells migrating in the wound area were taken at 10× magnification by Nikon Eclipse Ti microscope (Nikon, Tokyo, Japan). The quantitative values of the wound size were determined by web-based WimScratch module of Wimasis online software^44^.

### Cell invasion assay

Cell invasion assay was performed using BioCoat^TM^ Matrigel Invasion Chamber (6-well Boyden chamber) procured from BD Biosciences and were hydrated with Serum-free medium for 2 h prior to seeding of the cells. Briefly, cells were transfected with miR-195 or AntimiR-195 for 24 h and then serum-starved for 6 h. Following suspension in serum-free media, cells were introduced in top chambers (∼ 30,000 per well) and incubated at 37 °C, 5% CO_2_ incubator for 24 h. Lower chamber contains serum-loaded medium as attractant. Upon termination of the incubation, non invasive cells were removed carefully with a cotton swab and invasive cells were fixed with ethanol and stained using 0.05% Crystal Violet. After visualization through Nikon upright microscope, cells were lysed in 200 μL extraction buffer (Cell Biolab) and incubated for 10 min on an orbital shaker. Color developed thereafter was measured at 560 nM.

### Statistical analysis

Results are given as mean of 3 independent experiments ± SD. An independent Student’s two-tailed t test was performed using replicate values. Values of *P* < 0.05 were considered statistically significant.

## Additional Information

**How to cite this article**: Singh, R. *et al.* MicroRNA-195 inhibits proliferation, invasion and metastasis in breast cancer cells by targeting FASN, HMGCR, ACACA and CYP27B1. *Sci. Rep.* **5**, 17454; doi: 10.1038/srep17454 (2015).

## Supplementary Material

Supplementary Information

## References

[b1] Zhang M. H., Man H. T., Zhao X. D., Dong N. & Ma S. L. Estrogen receptor-positive breast cancer molecular signatures and therapeutic potentials. Biomed Rep. 2, 41–52 (2014). 24649067 10.3892/br.2013.187 PMC3916982

[b2] Zhang Y., Su H., Rahimi M., Tochihara R. & Tang C. EGFRvIII-induced estrogen-independence, tamoxifen-resistance phenotype correlates with PgR expression and modulation of apoptotic molecules in breast cancer. Int J Cancer. 125, 2021–2028 (2009). 19588487 10.1002/ijc.24540 PMC2855543

[b3] Currie E., Schulze A., Zechner R., Walther T. C. & Farese R. V. Cellular fatty acid metabolism and cancer. Cell Metab. 18, 153–161 (2013). 23791484 10.1016/j.cmet.2013.05.017 PMC3742569

[b4] Pizer E. S. *et al.* Inhibition of fatty acid synthesis induces programmed cell death in human breast cancer cells. Cancer Res. 56, 2745–2747 (1996). 8665507

[b5] Pizer E. S. *et al.* Inhibition of fatty acid synthesis delays disease progression in a xenograft model of ovarian cancer. Cancer Res. 56, 1189–1193 (1996). 8640795

[b6] Zheng L. *et al.* GDC-0941 sensitizes breast cancer to ABT-737 *in vitro* and *in vivo* through promoting the degradation of Mcl-1. Cancer Lett. 309, 27–36 (2011). 21664043 10.1016/j.canlet.2011.05.011

[b7] Bartel D. P. MicroRNAs: target recognition and regulatory functions. Cell. 136, 215–233 (2009). 19167326 10.1016/j.cell.2009.01.002 PMC3794896

[b8] Garzon R., Calin G. A. & Croce C. M. MicroRNAs in Cancer. Annu Rev Med. 60, 167–179 (2009). 19630570 10.1146/annurev.med.59.053006.104707

[b9] Chen Y. Q. *et al.* MicroRNA-195 promotes apoptosis in mouse podocytes via enhanced caspase activity driven by BCL2 insufficiency. Am J Nephrol. 34, 549–559 (2011). 22123611 10.1159/000333809

[b10] Singh R. & Saini N. Downregulation of BCL2 by miRNAs augments drug-induced apoptosis–a combined computational and experimental approach. J Cell Sci. 125, 1568–1578 (2012). 22328513 10.1242/jcs.095976

[b11] Bhattacharya A. *et al.* Regulation of cell cycle checkpoint kinase WEE1 by miR-195 in malignant melanoma. Oncogene. 32, 3175–3183 (2013). 22847610 10.1038/onc.2012.324

[b12] Lin Y. *et al.* Cyclin-dependent kinase 4 is a novel target in micoRNA-195-mediated cell cycle arrest in bladder cancer cells. FEBS Lett. 586, 442–447 (2012). 22289176 10.1016/j.febslet.2012.01.027

[b13] Xu T. *et al.* MicroRNA-195 suppresses tumorigenicity and regulates G1/S transition of human hepatocellular carcinoma cells. Hepatology. 50, 113–121 (2009). 19441017 10.1002/hep.22919

[b14] Yang G. *et al.* Upregulation of miR-195 increases the sensitivity of breast cancer cells to Adriamycin treatment through inhibition of Raf-1. Oncol Rep. 30, 877–889 (2013). 23760062 10.3892/or.2013.2532

[b15] Schiemann S., Schwirzke M., Brünner N. & Weidle U. H. Molecular analysis of two mammary carcinoma cell lines at the transcriptional level as a model system for progression of breast cancer. Clin Exp Metastasis. 16, 129–39 (1998). 9514094 10.1023/a:1021941203905

[b16] Kirschmann D. A., Seftor E. A., Nieva D. R., Mariano E. A. & Hendrix M. J. Differentially expressed genes associated with the metastatic phenotype in breast cancer. Breast Cancer Res Treat. 55, 127–36 (1999). 10481940 10.1023/a:1006188129423

[b17] Caton P. W., Holness M. J., Bishop-Bailey D. & Sugden M. C. PPARalpha-LXR as a novel metabolostatic signalling axis in skeletal muscle that acts to optimize substrate selection in response to nutrient status. Biochem J. 437, 521–530 (2011). 21609322 10.1042/BJ20110702

[b18] Kersten S., Desvergne B. & Wahli W. Roles of PPARs in health and disease. Nature. 405, 421–424 (2000). 10839530 10.1038/35013000

[b19] Lund E. G., Menke J. G. & Sparrow C. P. Liver X receptor agonists as potential therapeutic agents for dyslipidemia and atherosclerosis. Arterioscler Thromb Vasc Biol. 23, 1169–1177 (2003). 12615685 10.1161/01.ATV.0000056743.42348.59

[b20] Horton J. D. Sterol regulatory element-binding proteins: transcriptional activators of lipid synthesis. Biochem Soc Trans. 30, 1091–1095 (2002). 12440980 10.1042/bst0301091

[b21] Fielding C. J., Bist A. & Fielding P. E. Caveolin mRNA levels are up-regulated by free cholesterol and down-regulated by oxysterols in fibroblast monolayers. Proc Natl Acad Sci USA. 94, 3753–3758 (1997). 9108050 10.1073/pnas.94.8.3753 PMC20513

[b22] Murata M. *et al.* VIP21/caveolin is a cholesterol-binding protein. Proc Natl Acad Sci USA. 92, 10339–10343 (1995). 7479780 10.1073/pnas.92.22.10339 PMC40792

[b23] Ha J. *et al.* Cloning of human acetyl-CoA carboxylase cDNA. Eur J Biochem. 219, 297–306 (1994). 7905825 10.1111/j.1432-1033.1994.tb19941.x

[b24] Betel D., Wilson M., Gabow A., Marks D. S. & Sander C. The microRNA.org resource: targets and expression. Nucleic Acids Res. 36, D149–D153 (2008). 18158296 10.1093/nar/gkm995 PMC2238905

[b25] Kuhajda F. P. Fatty acid synthase and cancer: new application of an old pathway. Cancer Res. 66, 5977–5980 (2006). 16778164 10.1158/0008-5472.CAN-05-4673

[b26] Hunt D. A., Lane H. M., Zygmont M. E., Dervan P. A. & Hennigar R. A. mRNA stability and overexpression of fatty acid synthase in human breast cancer cell lines. Anticancer Res. 27, 27–34 (2007). 17352212

[b27] Chajes V., Cambot M., Moreau K., Lenoir G. M. & Joulin V. Acetyl-CoA carboxylase alpha is essential to breast cancer cell survival. Cancer Res. 66, 5287–5294 (2006). 16707454 10.1158/0008-5472.CAN-05-1489

[b28] Oliveras G. *et al.* Novel anti-fatty acid synthase compounds with anti-cancer activity in HER2+ breast cancer. Ann NY Acad Sci. 1210, 86–92 (2010). 20973802 10.1111/j.1749-6632.2010.05777.x

[b29] Orita H., Coulter J., Tully E., Kuhajda F. P. & Gabrielson E. Inhibiting fatty acid synthase for chemoprevention of chemically induced lung tumors. Clin Cancer Res. 14, 2458–2464 (2008). 18413838 10.1158/1078-0432.CCR-07-4177

[b30] Sinilnikova O. M. *et al.* Haplotype-based analysis of common variation in the acetyl-coA carboxylase alpha gene and breast cancer risk: a case-control study nested within the European Prospective Investigation into Cancer and Nutrition. Cancer Epidemiol Biomarkers Prev. 16, 409–415 (2007). 17372234 10.1158/1055-9965.EPI-06-0617

[b31] Lee J. S. *et al.* Fatty acid synthase inhibition by amentoflavone suppresses HER2/neu (erbB2) oncogene in SKBR3 human breast cancer cells. Phytother Res. 27, 713–720 (2013). 22767439 10.1002/ptr.4778

[b32] Mao J. H. *et al.* microRNA-195 suppresses osteosarcoma cell invasion and migration *in vitro* by targeting FASN. Oncol Lett. 4, 1125–1129 (2012). 23162665 10.3892/ol.2012.863 PMC3499598

[b33] Bjarnadottir O. *et al.* Targeting HMG-CoA reductase with statins in a window-of-opportunity breast cancer trial. Breast Cancer Res Treat. 138, 499–508 (2013). 23471651 10.1007/s10549-013-2473-6

[b34] Clendening J. W. & Penn L. Z. Targeting tumor cell metabolism with statins. Oncogene. 31, 4967–4978 (2012). 22310279 10.1038/onc.2012.6

[b35] Dunbar D. R. *et al.* Transcriptional and physiological responses to chronic ACTH treatment by the mouse kidney. Physiol Genomics. 40, 158–166 (2010). 19920212 10.1152/physiolgenomics.00088.2009 PMC2825763

[b36] Lopes N. *et al.* Alterations in Vitamin D signalling and metabolic pathways in breast cancer progression: a study of VDR, CYP27B1 and CYP24A1 expression in benign and malignant breast lesions. BMC Cancer. 10, 483 (2010). 20831823 10.1186/1471-2407-10-483 PMC2945944

[b37] Pfaffl M. W. A new mathematical model for relative quantification in real-time RT-PCR. Nucleic Acids Res. 29, e45 (2001). 11328886 10.1093/nar/29.9.e45 PMC55695

[b38] Chhabra R., Dubey R. & Saini N. Gene expression profiling indicate role of ER stress in miR-23a~27a~24-2 cluster induced apoptosis in HEK293T cells. RNA Biol. 8, 648–664 (2011). 21593605 10.4161/rna.8.4.15583

[b39] Adlakha Y. K. & Saini N. miR-128 exerts pro-apoptotic effect in a p53 transcription-dependent and -independent manner via PUMA-Bak axis. Cell Death Dis. 4, e542 (2013). 23492773 10.1038/cddis.2013.46 PMC3613825

[b40] Yadav V., Varshney P., Sultana S., Yadav, J. & Sain N. Moxifloxacin and ciprofloxacin induces S-phase arrest and augments apoptotic effects of cisplatin in human pancreatic cancer cells via ERK activation. BMC Cancer. 15, 581 (2015). 26260159 10.1186/s12885-015-1560-y PMC4531397

[b41] Adlakha Y. K. *et al.* Pro-apoptotic miRNA-128-2 modulates ABCA1, ABCG1 and RXRalpha expression and cholesterol homeostasis. Cell Death Dis. 4, e780 (2013). 23990020 10.1038/cddis.2013.301 PMC3763462

[b42] Chowdhari S. & Saini N. hsa-miR-4516 mediated downregulation of STAT3/CDK6/UBE2N plays a role in PUVA induced apoptosis in keratinocytes. J Cell Physiol. 229, 1630–1638 (2014). 24610393 10.1002/jcp.24608

[b43] Yadav V., Sultana S., Yadav J. & Saini N. Gatifloxacin induces S and G2-phase cell cycle arrest in pancreatic cancer cells via p21/p27/p53. PLoS One. 7, e47796 (2012). 23133524 10.1371/journal.pone.0047796 PMC3485023

[b44] Goel A. *et al.* DAMTC regulates cytoskeletal reorganization and cell motility in human lung adenocarcinoma cell line: an integrated proteomics and transcriptomics approach. Cell Death Dis. 3, e402 (2012). 23059821 10.1038/cddis.2012.141 PMC3481129

