# Supplementary material for: MicroRNA-195 inhibits proliferation, invasion and metastasis in breast cancer cells by targeting FASN, HMGCR, ACACA and CYP27B1
Source: Sci Rep. 2015 Dec 3;5:17454. doi: 10.1038/srep17454 (PMC4668367; doi:10.1038/srep17454)
Supplement: Supplementary Information [file srep17454-s1.pdf]

MicroRNA-195 inhibits proliferation, invasion and metastasis in breast cancer cells by targeting FASN, HMGCR, ACACA and CYP27B1

Richa Singh[1], Vikas Yadav[1], Sachin kumar[1], Neeru Saini[1 *]

[1]Functional Genomics Unit, CSIR-Institute of Genomics and Integrative Biology (IGIB). Council of Scientific & Industrial Research (CSIR), Delhi, India.

Supplementary Figure S1

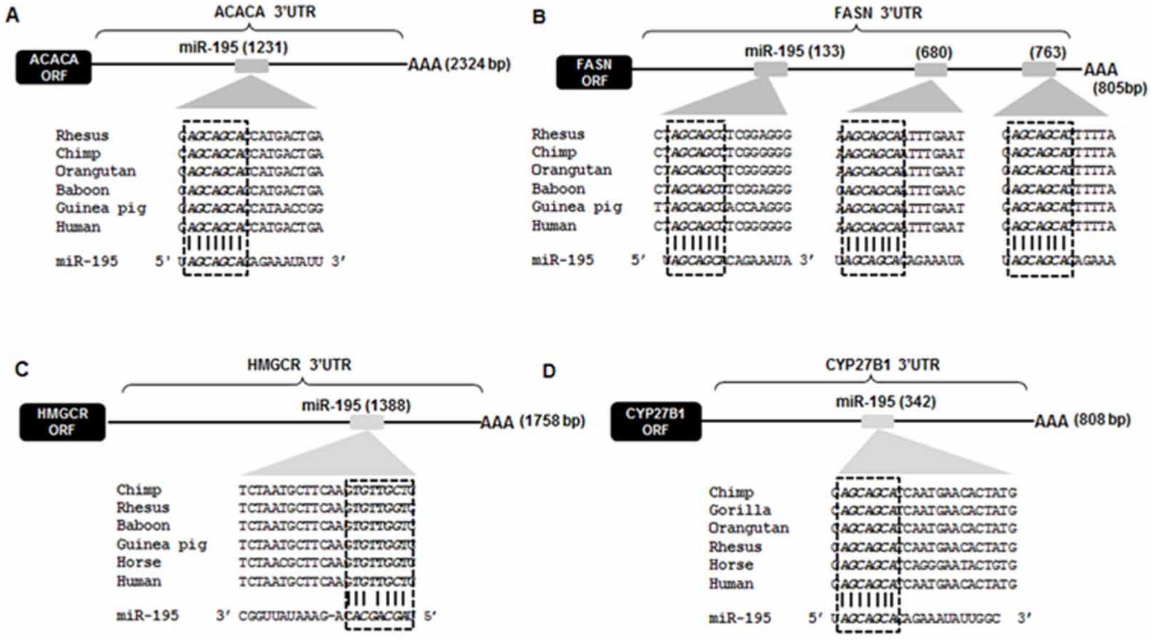

Predicted binding site of miR-195 in FASN, HMGCR, ACACA, CYP27B1

Supplementary Figure S2

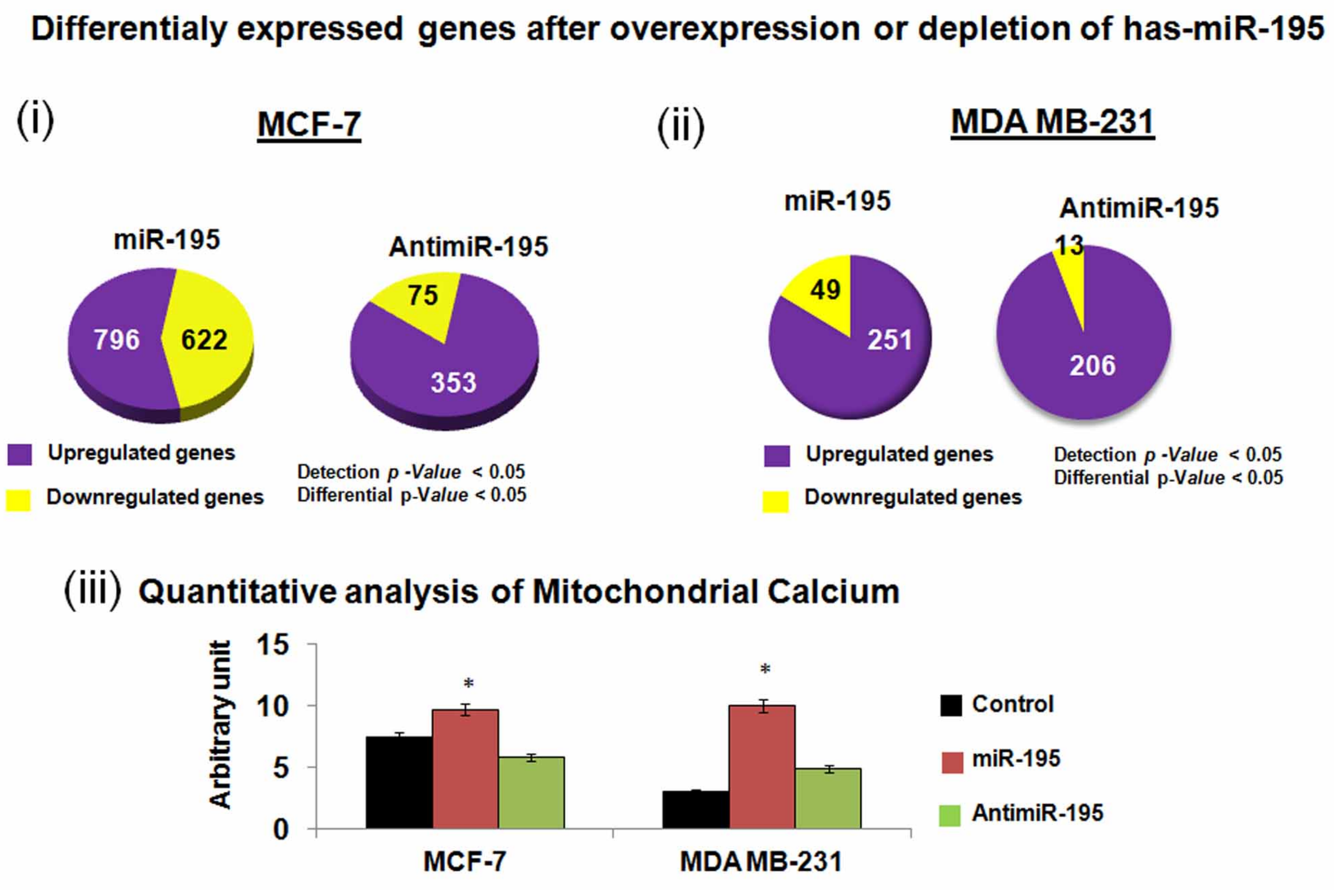

Supplementary Figure S3

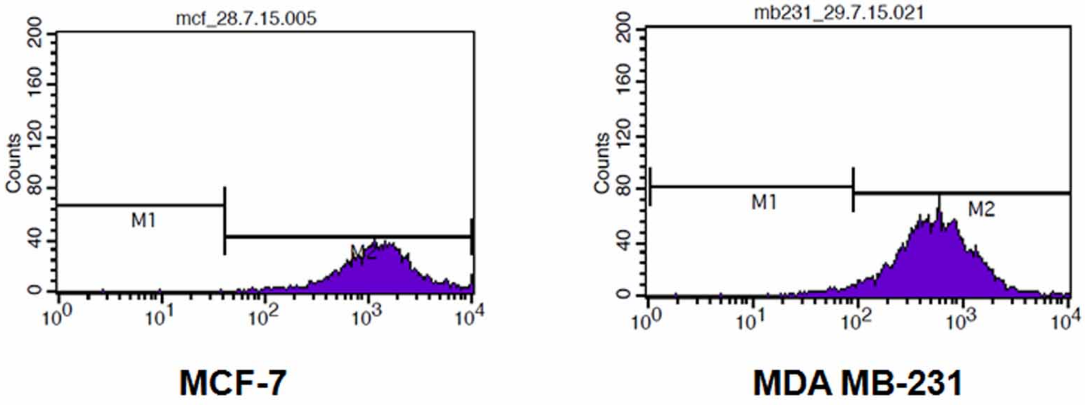

Region M1 (Day 0) has been gated to show the percentage of proliferated cells at Day 2 and is represented in the form of bar graph in Figure 7A.

Supplementary Figure S4

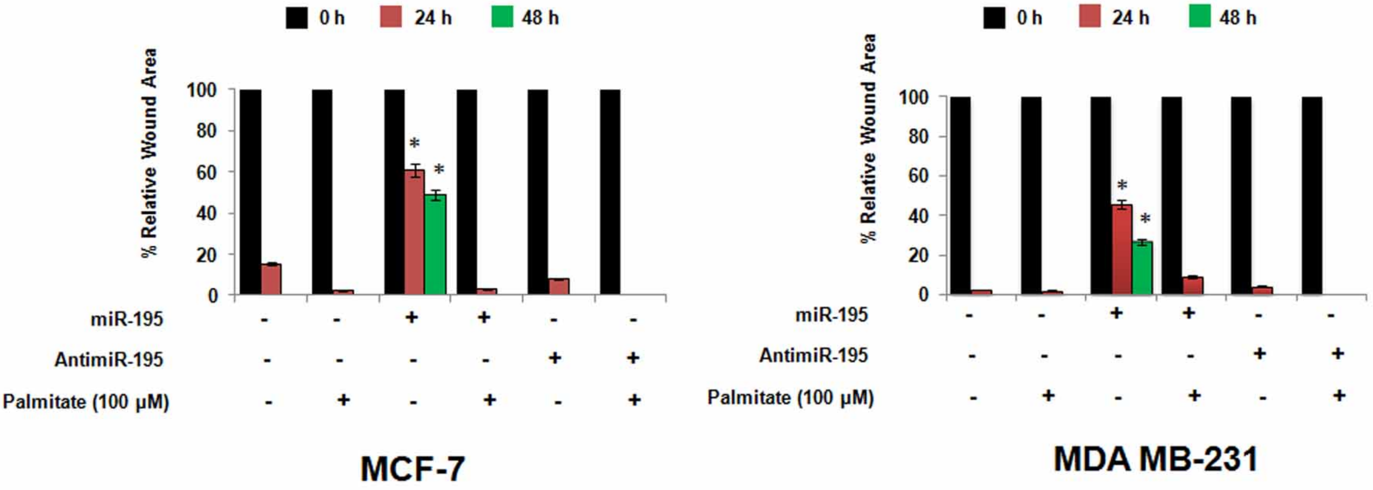

Quantitaive analysis of Wound Healing assay: Graphical representation of the quantitative values of the wound / scratch size after miR-195 and AntimiR transfection in presence or absence of Palmitate at different time points as determined by the WimScratch module of Wimasis online software. The bar diagram represent mean ± S.D for three independent experiments. * p < 0.01

Supplementary Figure S5

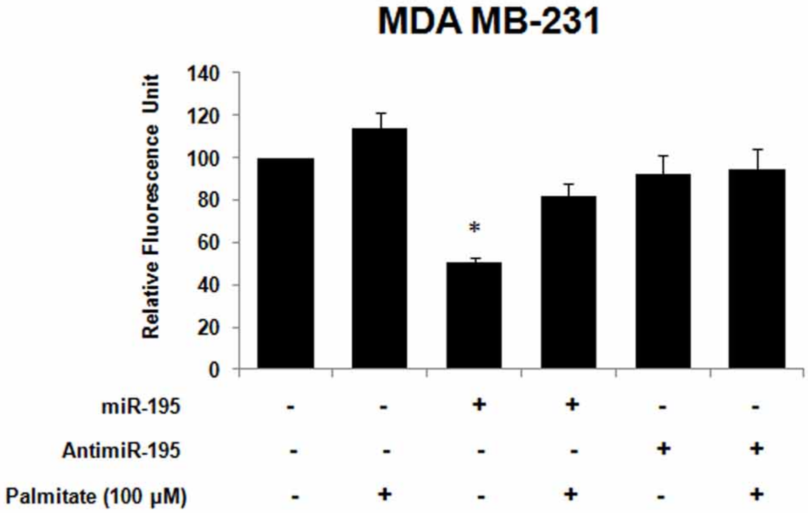

Quantitative analysis of Invasion assay: Graphical representation of the quantitative values of the invasion assay as determined by the colorimetric analysis using cell lytic buffer and further reading at 550nM. The bar diagram represent mean ± S.D for three independent experiments. * $p < 0.01$

SUPPLEMENTARY INFORMATION

MicroRNA-195 attenuates Epithelial-Mesenchymal-Transition (EMT) in breast cancer cells by targeting FASN, HMGCR, ACACA and CYP27B1

Richa Singh, Vikas Yadav, Sachin Kumar and Neeru Saini.

Supplementary Table S1: List of primers used in the present study

**18S rRNA FP**	GTAACCCGTTGAACCCCATT
**18S rRNA RP**	CCATCCAATCGGTAGTAGCG
**PGC-1α FP**	TGCCCT GGATTGTTGACATGA
**PGC-1α RP**	TTTGTCAGGCTGGGGGTAGG
**PPARα FP**	GGCGAGGATAGTTCTGGAAGC
**PPARα RP**	CACAGGATAAGTCACCGAGGAG
**LXRα FP**	GTTATAACCGGGAAGACTTTGCCA
**LXRα RP**	GCCTCTCTACCTGGAGCTGGT
**LXRβ FP**	CGTGGACTTCGCTAAGCAAGTG
**LXRβ RP**	GGTGGAAGTCGTCCTTGCTGTAGG
**SREBP2 FP**	AGGAGAACATGGTGCTGA
**SREBP2 RP**	TAAAGGAGAGGCACAGGA
**SREBP1 FP**	GCAAGGCCATCGACTACATT
**SREBP1 RP**	GGTCAGTGTGTCCTCCACCT
**HMGCS1 FP**	TCCCACTCCAAATGATGACA
**HMGCS1 RP**	CTTCAGGTTCTGCTGCTGTG
**IDI1 FP**	GCATCGAGCTTTTAGTGTCTTCT
**IDI1 RP**	GGCTGGATTGCTTAATGGATGA
**LDLR FP**	GTCTTGGCACTGGAACTCGT
**LDLR RP**	CTGGAAATTGCGCTGGAC
**ABCA1 FP**	GCACTGAGGAAGATGCTGAAA
**ABCA1 RP**	AGTTCCTGGAAGGTCTTGTTCAC
**ABCG1 FP**	CAGGAAGATTAGACACTGTGG
**ABCG1 RP**	GAAAGGGGAATGGAGAGAAGA
**ABCG5 FP**	ACCCAAAGCAAGGAACGGGAA
**ABCG5 RP**	CAGCGTTCAGCATGCCTGTGT
**NR1I3 FP**	AGTTGCACAGGTGTTTGCTG
**NR1I3 RP**	GTGCTTAGATGCTGGCATGA
**CYP2B6 FP**	ATGGGGCACTGAAAAAGACTGA
**CYP2B6 RP**	AGAGGCGGGGACACTGAATGAC
**ACACA FP**	AATCTTGAGGGCTAGGTCTTTCTGGA
**ACACA RP**	CCAGAGGTTGGGCCAAGGGA
**FASN FP**	CAGAGTCGGAGAACTTGCAG
**FASN RP**	GGAGGCATCAAACCTAGACAG
**HMGCR FP**	TCGGTGGCCTCTAGTGAGAT
**HMGCR RP**	TGTCCCCACTATGACTTCCC
**CYP27B1 FP**	TGGCCCAGATCCTAACACATTT
**CYP27B1 RP**	GTCCGGGTCTTGGGTCTAACT

Supplementary Table S2: The list of 1418 differentially expressed genes after over-expression of hsa-miR-195 in MCF7 cells

GENE	FOLD CHANGE	GENE	FOLD CHANGE	GENE	FOLD CHANGE	GENE	FOLD CHANGE
AAMP	1.406722	AP4E1	1.603214	BBS2	0.599011	C15ORF39	1.448212
AARSD1	1.896856	APBA3	1.89414	BCKDK	0.665757	C15ORF42	1.932431
AATF	1.58622	APP	0.651712	BCL11A	0.71586	C16ORF48	1.68059
ABHD14A	0.687401	AQP11	0.454974	BCL3	2.634313	C16ORF52	0.649889
ACBD6	1.384792	ARC	3.395515	BCYRN1	3.042853	C16ORF68	1.854429
ACD	1.423573	ARID3B	1.443875	BEX4	0.562305	C16ORF7	1.920338
ACIN1	1.492325	ARL1	0.687927	BIRC6	1.454864	C16ORF74	0.562434
ACSM3	0.565535	ARL16	1.75541	BMI1	0.657751	C16ORF75	0.656651
ACTA1	2.806901	ARL2	0.646104	BNC2	0.641357	C17ORF67	1.672934
ACTR6	0.704585	ARL6IP5	0.689749	BNIP3L	0.607638	C19ORF22	1.554116
ADAL	0.629874	ARMC6	1.442871	BP75	0.580623	C19ORF29	1.735426
ADAMTS1	0.672067	ARPC1B	0.482248	BRAF	2.629773	C19ORF30	2.941134
ADAP2	0.564443	ARSK	0.59205	BRPF1	1.597267	C19ORF40	0.554032
ADCK1	1.638868	ARV1	0.716383	BSN	2.588085	C19ORF42	0.663684
ADH5	0.432273	ASB13	0.600652	BTF3L4	0.51966	C1ORF102	1.973908
ADORA2B	0.587941	ASB9	0.412629	BUD13	1.682624	C1ORF162	2.228043
ADRM1	1.497022	ASCC2	1.454681	BUD31	1.339298	C1ORF182	0.479156
AEN	2.927039	ASGR1	0.538345	C10ORF114	0.442027	C1ORF50	1.693135
AGBL5	1.232442	ATAD3A	2.089899	C10ORF137	1.290311	C1QBP	0.645912
AHI1	1.568372	ATF7IP2	1.812408	C10ORF41	0.318084	C1RL	0.501254
AHSA2	1.325811	ATG2A	1.65495	C10ORF57	0.657055	C20ORF55	1.640115
AIM1L	1.895868	ATL2	0.714907	C10ORF75	0.524827	C2ORF37	2.814006
AKR1C3	0.617817	ATL3	1.406718	C10ORF84	1.420229	C2ORF64	0.636466
AKT1S1	1.578531	ATP13A1	1.496007	C11ORF54	0.69112	C2ORF68	0.526649
ALDH1A3	0.657309	ATP1B1	0.733385	C11ORF57	1.451817	C2ORF82	2.773965
ALDOC	0.491504	ATP5L	0.806702	C11ORF75	0.644749	C3ORF1	0.452014
ALG1L	0.346398	ATP6V1B1	1.895603	C12ORF44	1.9078	C3ORF10	0.430126
ALPL	0.499451	ATPBD1B	1.49821	C12ORF76	0.577882	C3ORF19	1.736321
AMOT	0.567166	ATRIP	1.585634	C13ORF25	0.48334	C3ORF58	0.568912
ANGEL1	1.618432	ATXN2	1.322884	C13ORF34	1.3049	C3ORF63	0.586924
ANKHD1-	1.530376	AUH	0.509428	C14ORF124	0.34254	C4ORF31	0.668713
ANKRD1	8.32418	AUTS2	0.646816	C14ORF126	0.286153	C4ORF49	0.524672
ANKRD26	1.64055	AXUD1	2.107915	C14ORF132	0.620519	C5ORF13	0.567786
ANKRD43	0.633131	B3GALNT2	2.03693	C14ORF147	0.521529	C5ORF21	0.737093
ANKRD46	0.764499	B3GALT6	0.701475	C14ORF2	0.668548	C5ORF41	1.78813
ANKRD47	2.350186	B3GALTL	0.507887	C14ORF32	0.470897	C5ORF5	1.97771
ANKRD54	2.725137	B9D1	0.628589	C14ORF37	0.55312	C5ORF51	1.350569
ANKRD57	0.694718	BAPX1	0.548013	C14ORF79	2.60753	C6ORF111	0.685148
AP1S2	0.668466	BAT5	1.511187	C14ORF93	0.608094	C6ORF117	0.536495
AP3S1	0.534897	BBS10	0.550494	C15ORF23	0.478011	C6ORF124	0.547975
C6ORF130	0.630575	CCNH	0.588365	CLK1	1.949995	DCAF16	0.602283
C6ORF162	0.575501	CCRK	1.723311	CLN6	1.5503	DCP1A	1.680696
C6ORF52	2.823737	CCRN4L	1.362913	CMAS	0.625394	DCP2	1.435504

GENE	FOLD CHANGE	GENE	FOLD CHANGE	GENE	FOLD CHANGE	GENE	FOLD CHANGE
C6ORF64	0.552607	CCT4	0.592406	CNIH4	1.260059	DDB2	2.455964
C6ORF72	0.521022	CCT5	0.734152	CNOT10	1.379755	DDTL	0.497385
C6ORF85	0.591246	CD19	0.535202	CNOT3	1.919268	DDX10	1.433616
C7ORF40	2.668501	CD46	0.508468	CNPY3	0.571485	DDX23	1.307363
C7ORF63	3.15595	CD58	0.508406	CNTNAP2	0.686529	DDX39	1.459288
C9ORF102	1.677677	CDC23	0.771392	COG4	1.62857	DDX51	2.052425
C9ORF123	0.632665	CDC25C	0.719622	COL13A1	0.589547	DDX56	1.473939
C9ORF142	1.311945	CDC42SE1	0.714017	COL5A1	0.662915	DEPDC6	0.476304
C9ORF58	0.728109	CDC5L	1.528963	COMMD1	1.462526	DEXI	0.59804
C9ORF75	1.485273	CDCA2	1.483393	COPA	1.29658	DFNA5	0.654241
C9ORF86	1.371992	CDS2	0.731777	COPS7B	0.768617	DHDH	1.64153
C9ORF90	1.328337	CELSR3	2.420456	COQ10A	0.607801	DHRS2	2.815501
C9ORF98	2.319595	CENPK	0.472205	COQ3	0.761368	DHX29	1.691183
CA12	0.448296	CENPT	1.463273	COQ7	0.555459	DIS3L2	1.936821
CACNA2D3	2.166661	CEP250	2.800675	CORO1A	1.776072	DKFZP686	0.472571
CADM1	0.649042	CEP350	1.491231	CP110	1.370297	DKK3	0.368502
CAMK2N1	0.730165	CEP70	0.660268	CPE	0.625645	DLD	0.480064
CASC1	4.390188	CGGBP1	1.081219	CPLX1	2.338235	DLST	2.269375
CASC4	0.367054	CGNL1	0.687615	CPVL	0.553639	DLX1	0.472438
CASK	0.72287	CHCHD6	1.442264	CREG1	0.474797	DMWD	2.066703
CAV2	0.516536	CHD1	1.458014	CRIP2	1.868389	DNAH1	1.774604
CBL	1.710471	CHD8	1.820413	CRYZ	0.663774	DNAJB2	2.236587
CBR4	0.593676	CHERP	1.628274	CSPG5	0.576746	DNAJC25	1.861884
CCDC109B	0.763315	CHFR	1.362785	CTDP1	1.690435	DNASE1	1.787928
CCDC110	0.633802	CHMP1A	1.932658	CTGLF3	1.720175	DNASE2	0.607129
CCDC16	0.65564	CHST12	0.687896	CTSC	0.661355	DOCK11	0.595062
CCDC45	1.537272	CHST13	0.731548	CTSL1	0.697624	DOCK2	0.5539
CCDC66	2.184493	CHST15	0.738942	CTSL2	0.631252	DOHH	2.262024
CCDC76	1.705059	CHSY3	0.696894	CTXN1	0.693749	DPF1	1.858416
CCDC81	2.105132	CHURC1	0.473425	CXCR7	0.44257	DPP9	1.911881
CCDC84	1.49162	CISD2	0.606296	CYP1B1	0.486747	DPPA2	0.51045
CCDC86	2.441578	CIZ1	2.501085	CYP26B1	0.504953	DPYSL2	0.662593
CCDC92	0.592145	CKMT1A	1.366663	CYP27B1	0.496069	DPYSL3	0.734442
CCDC93	1.643709	CLCN6	2.042817	CYP3A5	2.510812	DRAP1	1.385436
CCHCR1	1.574622	CLCN7	1.360879	CYP4V2	0.533607	DRG2	1.705488
CCNB1	0.664886	CLDN12	0.638925	DAPK3	3.233264	DSCR6	0.636932
CCND2	0.715093	CLEC4A	1.938707	DAXX	1.536893	DSTYK	1.573678
DUS3L	2.214824	ETFDH	0.663428	FGFBP1	0.356532	GHR	0.53466
DUSP14	1.475088	ETV5	2.097342	FKBP11	1.451644	GLCE	0.536792
DUSP5	2.287305	EWSR1	1.24505	FKBP15	1.787784	GLI2	0.594422
DUSP8	2.528717	EXOC3	1.419449	FLAD1	1.562036	GLIPR2	0.720553
DUT	0.628473	EXOSC9	1.485968	FLJ10996	1.932474	GLTPD1	1.845348
DVL2	1.505043	EXTL2	0.621282	FLJ12684	0.48117	GMDS	0.701222
DYNLRB2	3.050456	F12	0.624572	FLJ12949	1.534043	GNA11	1.366573
E2F5	0.455319	FADS1	0.720107	FLJ20254	1.57968	GNG4	1.488706
E2F7	1.553749	FAM100B	1.638305	FLJ20699	1.519543	GNGT1	3.632056

GENE	FOLD CHANGE	GENE	FOLD CHANGE	GENE	FOLD CHANGE	GENE	FOLD CHANGE
E4F1	1.454523	FAM105A	0.601354	FLJ22222	0.525444	GNL2	1.387598
EBPL	0.549404	FAM111A	1.490539	FLJ22795	1.798417	GNS	0.760744
ECH1	0.690832	FAM122B	0.61617	FLJ31568	3.062702	GOLGB1	1.765122
ECSIT	1.911805	FAM133B	1.636534	FLJ36070	0.494271	GOLSYN	0.538541
EDA2R	2.230575	FAM136A	0.684116	FMO4	0.60491	GORASP1	1.501606
EDC3	1.882379	FAM13B	2.006295	FNBP4	1.639547	GPC3	0.416085
EEA1	2.093785	FAM158A	2.488572	FOXA1	0.625313	GPC4	0.589511
EEF1A1	0.919772	FAM162B	0.470448	FOXC1	0.646203	GPER	0.366541
EFNB2	0.775993	FAM174B	0.465937	FOXQ1	0.634412	GPKOW	2.202312
EFTUD1	1.595679	FAM188B	2.043956	FRAG1	0.688163	GPM6B	0.580409
EFTUD2	1.32464	FAM193A	2.166015	FRMD4A	0.511268	GPR160	0.602603
EIF2A	0.629862	FAM27A	2.614315	FTH1	0.650423	GPR172A	1.783163
EIF3B	1.291229	FAM39E	1.370896	FXC1	0.578511	GPR37	0.452553
EIF3D	1.4439	FAM3A	1.629127	FXR2	1.800094	GPR64	0.563584
EIF3J	0.58836	FAM3C	0.434539	FYCO1	0.725026	GPS2	1.386234
EIF4G3	1.42198	FAM40A	1.625073	FZD4	0.666153	GRN	0.62206
ELAC2	1.336368	FAM73B	1.413266	GABBR2	1.823239	GRWD1	1.43184
ELMOD1	0.44142	FAM80B	3.753154	GAD1	1.415783	GSTA1	0.32194
ENDOD1	0.658258	FAM84B	0.535154	GADD45B	1.670468	GSTA4	0.679501
ENO3	1.849094	FAM98A	1.426336	GADD45G	2.902838	GSTM2	0.670848
EPC1	1.529821	FANCI	0.674161	GALK1	0.782677	GTF2F1	1.319513
EPHA4	0.435988	FARS2	1.334646	GALNT12	0.574917	GUSBL1	1.511526
EPHB1	0.666656	FARSA	1.926094	GALR2	3.844585	HADHA	0.668705
ERAL1	1.38189	FBRS	1.555545	GAS1	0.414953	HADHB	0.655517
ERAP2	0.675102	FBXL11	1.487263	GAS8	2.335095	HAS2AS	0.556512
ERCC2	1.483063	FBXL20	1.49095	GBF1	1.669184	HBEGF	1.711633
ERCC3	1.320362	FBXL21	0.581844	GBGT1	0.559074	HBQ1	0.51285
ERF	1.410842	FBXL6	2.42629	GBP2	0.417074	HCCA2	1.611028
ERP29	0.667853	FBXW4	1.521124	GCA	0.608757	HCFC1	0.603492
ERRFI1	0.643998	FDX1L	1.783172	GCAT	1.624292	HCFC2	2.200684
ESD	0.610381	FGD3	0.508315	GGT7	1.919797	HDHD1A	0.644578
HELZ	1.437119	IGSF3	0.770882	KCTD20	0.749811	LINCR	3.440799
HESX1	0.473236	IGSF5	0.516802	KDM5B	1.657634	LIPA	0.478548
HEY1	0.464445	IL28RA	1.43864	KDM6B	1.842935	LITAF	0.462146
HEY2	1.509004	IL32	5.74988	KIAA0133	1.375401	LMBRD1	0.513758
HGS	1.327428	ILKAP	2.423556	KIAA0194	1.537265	LMF2	1.59457
HIST1H1C	2.219726	IMPA2	0.549209	KIAA0240	0.615361	LMNB2	1.312919
HIST1H2AM	2.239455	INO80	1.872553	KIAA0367	0.513271	LMTK2	1.8806
HIST1H4H	7.353714	INO80D	1.62208	KIAA0562	1.417314	LOC100128252	0.606457
HIST1H4K	3.565747	INPP5E	1.742749	KIAA0586	1.646999	LOC100128274	2.791503
HIST2H2AA4	2.165785	INPP5K	1.500102	KIAA0753	2.161771	LOC100128805	1.633569
HIST2H2AC	2.25678	INSIG2	0.697945	KIAA0907	1.361119	LOC100129028	1.484395
HKR1	1.325407	INTS8	1.573714	KIAA0913	1.496151	LOC100129637	1.739444
HLA-DMB	0.721075	IP6K1	1.443672	KIAA1285	1.548919	LOC100130886	0.705399
HLA-DPA1	0.391707	IPO13	1.906653	KIAA1429	1.491355	LOC100131205	0.393905
HLA-DRB1	0.564605	IPO8	0.624202	KIAA1467	0.621426	LOC100131609	0.554123

GENE	FOLD CHANGE	GENE	FOLD CHANGE	GENE	FOLD CHANGE	GENE	FOLD CHANGE
HMGN4	0.583111	IQCC	1.998744	KIAA1683	2.124963	LOC100132112	2.12007
HMHA1	0.588091	IQCK	1.719204	KIAA1688	1.763008	LOC100132299	0.666789
HMOX1	1.787614	IRAK2	1.678422	KIAA1731	3.078803	LOC100132418	1.706062
HNRNPA1	0.430211	IRS2	0.449895	KIF24	2.1473	LOC100132442	0.385049
HNRPA1L-2	0.708533	IRX3	0.688911	KIF2C	1.294545	LOC100132457	1.555875
HNRPA1P4	0.726899	IRX4	0.627465	KIF7	1.774129	LOC100132564	7.190289
HNRPDL	1.193824	IRX6	1.606796	KIT	0.546363	LOC100132774	1.963326
HOXB8	0.601496	ISG15	2.61837	KLF13	0.645203	LOC100133012	0.522178
HP1BP3	1.506239	ISG20L1	2.194036	KLF9	0.588001	LOC100133372	0.509126
HPRT1	0.6529	ISL1	0.664507	KLHL18	1.604039	LOC100133489	0.509144
HRAS	1.471732	ISOC1	0.645349	KLHL28	2.683914	LOC100133866	1.799959
HSBP1	0.528112	ITGAV	0.619936	KPNA1	1.551411	LOC100134108	1.792875
HSD17B6	0.365109	IVD	1.440399	KPNA4	1.586982	LOC100134134	0.744554
HSD17B8	0.645334	IWS1	1.651962	KRCC1	1.442211	LOC100134304	0.656216
HSPB8	1.623281	JAKMIP2	0.617354	KRI1	1.714236	LOC100134364	1.441668
HSPBL2	0.702746	JARID1A	1.5711	LACTB2	0.460349	LOC144438	1.4921
IARS2	0.687828	JHDM1D	2.901421	LAMC3	0.591391	LOC196752	1.951707
ID2	0.649242	JMJD1A	1.928174	LANCL1	0.633911	LOC257396	0.457961
IER3	2.435408	JMJD2C	1.823865	LARP7	0.650737	LOC285908	2.484783
IER5L	3.676952	JUNB	1.87051	LEMD2	1.317535	LOC286208	2.485033
IFI30	1.517129	KAL1	0.557853	LGI2	0.576897	LOC387882	0.668762
IFI35	0.674457	KBTBD8	1.976402	LHPP	0.564549	LOC388275	0.594542
IFIT2	3.6234	KCNJ8	0.718002	LHX2	0.769839	LOC388339	1.486259
IFT74	1.684502	KCNK12	2.73258	LIME1	1.759477	LOC388789	0.659408
IGFBP5	0.423895	KCNMB4	0.747103	LIN37	2.796925	LOC388796	3.222598
LOC389156	0.365518	LOC650803	1.424483	LRRC3	0.522559	MKI67	1.526061
LOC399491	2.101416	LOC651149	0.482854	LRRC42	0.501652	MKX	0.568641
LOC400027	0.702357	LOC651816	1.397898	LTA	2.442293	MLL	1.842015
LOC401152	0.544216	LOC653156	0.37231	LTA4H	0.724283	MMD	0.723756
LOC401317	2.225312	LOC653171	0.553252	LTBP2	0.441152	MOBKL2B	0.584672
LOC401397	0.511822	LOC653344	1.7901	LTBR	1.385414	MORF4L1	0.499784
LOC401640	0.512936	LOC653610	2.212405	LXN	0.558148	MORG1	1.418072
LOC402221	0.647847	LOC727820	2.238738	LYG1	2.4697	MPP3	1.556841
LOC439949	0.574018	LOC728153	1.699024	LZTFL1	1.436195	MPP6	0.613993
LOC440354	3.74157	LOC728308	2.022071	MAD1L1	2.568147	MRPL10	1.840231
LOC440928	0.574572	LOC728417	2.129579	MAF1	1.400931	MRPL12	1.258646
LOC441511	0.406131	LOC728640	1.580736	MAGOHB	0.509783	MRPL21	1.094569
LOC441642	0.591579	LOC728643	0.417828	MAL2	0.606453	MRPL38	1.458246
LOC441876	0.759792	LOC728661	0.75893	MAOA	0.626184	MRPS15	1.281546
LOC442582	0.533607	LOC728741	2.64831	MAP1S	1.581289	MRPS2	1.430535
LOC493869	0.72112	LOC728820	0.752857	MAP2K2	1.779221	MRPS27	0.671561
LOC641849	0.326562	LOC728823	1.553641	MAP3K1	1.43825	MRPS5	1.306663
LOC642590	0.714751	LOC728908	1.6042	MAP3K11	1.629348	MTCH2	0.606467
LOC642826	3.166244	LOC729102	0.776851	MAP3K9	2.34105	MTHFD2L	2.072323
LOC642934	0.718439	LOC729148	0.699451	MAP7D2	0.598711	MTHFR	1.809215
LOC643007	0.661588	LOC729421	0.549204	MAPK13	0.619819	MTIF3	0.619655

GENE	FOLD CHANGE	GENE	FOLD CHANGE	GENE	FOLD CHANGE	GENE	FOLD CHANGE
LOC643201	2.709609	LOC729608	1.299458	MCCC1	1.370606	MTRF1L	1.555912
LOC644033	1.486373	LOC730153	2.117907	MCL1	1.507677	MUC16	4.998588
LOC644132	1.858754	LOC730173	1.823567	MCM2	1.567329	MUM1	0.769184
LOC644879	1.594988	LOC730246	0.773362	MCM5	2.281943	MURC	2.183013
LOC644928	1.617436	LOC730256	2.109903	MDM4	0.440064	MUS81	1.742258
LOC645385	0.654099	LOC731314	1.387617	MEA1	1.759348	MYBBP1A	1.986199
LOC645436	0.517202	LOC731797	0.414758	MED14	0.694291	MYC	0.310825
LOC645937	1.689481	LOC731950	1.576763	MED4	0.717791	MYCBP2	1.588026
LOC645968	0.523999	LOC732432	0.463795	METAP2	0.730529	MYH3	2.393872
LOC646332	2.933125	LOC81691	1.458091	METTL4	0.578015	MYL5	1.516999
LOC646347	0.704743	LOC88523	1.722571	METTL5	0.564554	MYO3A	1.913228
LOC646576	0.391364	LOC92497	1.686935	MFSD3	0.569149	NAB2	1.410995
LOC646786	1.992027	LPAR3	0.443546	MGC16169	1.658559	NADSYN1	1.36938
LOC647349	0.399789	LPL	0.725003	MGC16703	1.249777	NAIF1	2.115981
LOC648210	0.490154	LPPR2	1.648397	MGC3020	1.526591	NAPB	1.383741
LOC648984	1.987215	LPXN	2.173012	MGC52000	1.994033	NAPG	1.457612
LOC649839	1.939873	LRIG2	1.722567	MGMT	0.682238	NARFL	1.682377
LOC650029	1.67466	LRIG3	0.518663	MICALL1	1.682829	NAT10	1.326421
LOC650298	1.508364	LRP5L	3.706272	MIRLET7D	0.42192	NCAPD3	1.300616
NCAPG	1.367319	NUP214	1.961623	PDX1	1.81116	PPAN	1.698934
NCKAP1	0.446588	NUP37	0.635217	PDXDC1	0.698987	PPAP2A	0.693017
NCOA5	1.612944	OAZ2	1.447161	PEA15	1.4769	PPIA	0.527531
NDUFB9	1.288156	ODF2	1.442259	PECR	0.727763	PPIC	0.56449
NEK11	2.231478	OFD1	1.913966	PELP1	2.026943	PPP1CB	0.516429
NEK2	1.366881	OIP5	0.557956	PEX14	1.731047	PPP1R12C	2.075874
NELF	1.448877	OKL38	3.623806	PGBD5	1.741494	PPP1R14C	0.66949
NELL2	0.700182	OSAP	0.619581	PGD	0.781266	PPP1R15A	2.162491
NETO2	0.657455	OSBPL1A	0.656703	PGS1	1.448716	PPP2R5B	2.268868
NEU1	1.578082	OSGEPL1	0.425931	PHC3	1.894499	PPP3CB	0.610261
NEUROG2	0.650746	OVGP1	3.383078	PHF21B	4.535304	PPPDE1	0.761485
NEXN	2.025849	OXR1	0.732317	PHIP	1.711606	PPRC1	1.545904
NFKB1	1.807571	OXSR1	1.379797	PHLDA3	2.199798	PQLC3	0.690366
NFKBIA	1.286472	OXTR	1.336925	PIAS3	1.563056	PRCP	0.729331
NFKBIB	2.691425	PA2G4	1.358544	PIGF	0.517208	PRDM10	0.529069
NFKBIL2	2.250907	PACSIN1	0.656573	PIGV	0.67615	PRDX6	0.670402
NFRKB	1.706325	PAF1	1.436774	PIGY	0.620511	PRKAB1	1.520015
NHP2L1	0.621167	PAFAH1B1	0.558365	PIK3IP1	2.756854	PRKCABP	1.592081
NIF3L1	0.653868	PAFAH1B3	0.644289	PIK3R4	1.394981	PRKCH	0.720208
NKD2	1.670086	PAM	0.769178	PIP4K2A	0.721969	PRKRIP1	1.595104
NKTR	1.482431	PAPD5	1.369272	PIR	0.575784	PRMT10	1.616779
NLE1	2.055714	PAQR8	0.587375	PISD	1.401983	PROS1	0.743147
NOC2L	1.637139	PARM1	0.665399	PLA2G4C	9.909851	PRPF19	1.347475
NOC3L	1.499519	PARP6	2.117924	PLA2G7	0.353809	PRPF3	1.453293
NOL6	1.559265	PATL1	1.367563	PLAC8L1	2.079184	PRR4	4.038555
NOL8	1.355428	PAX6	0.538198	PLD6	2.010165	PRR7	1.796886
NOP14	1.427678	PBK	0.640708	PLEKHF1	1.401021	PRRG1	0.660595

GENE	FOLD CHANGE	GENE	FOLD CHANGE	GENE	FOLD CHANGE	GENE	FOLD CHANGE
NOP2	1.428566	PBX3	0.647446	PLEKHM2	1.969477	PRRG2	0.522795
NPIP	1.722996	PBX4	1.919902	PLS1	0.675394	PRRX2	0.70185
NPY	0.484033	PCGF1	2.058911	PLSCR3	0.643035	PRSS16	0.516173
NR1H3	2.029132	PCMT1	0.75387	PMS2L3	2.408704	PRSS23	0.511874
NR2F1	0.599125	PCMTD2	0.66596	PNMA2	0.642966	PRSS8	0.35762
NSUN6	1.585816	PCOLCE2	0.55933	POFUT2	1.928212	PSAP	0.776054
NT5C2	1.552747	PCSK1N	0.59688	POLA2	1.277775	PSEN2	1.477739
NUBP1	0.601414	PCSK7	1.580344	POLE	1.511246	PSMD3	1.757958
NUCB1	0.784487	PDCD2L	1.365747	POLM	2.300695	PTBP2	1.776606
NUDT11	0.686473	PDGFD	0.514107	POLR2A	1.539453	PTCD2	1.330105
NUDT16	0.546196	PDGFRL	0.681412	POLR3A	1.800054	PTCH1	0.438583
NUDT16L1	1.732993	PDHB	0.619454	POLR3D	1.708613	PTDSS2	1.701543
NUDT7	0.590328	PDIA6	0.633673	PON2	0.547596	PTGER1	0.484542
PTGER2	0.39034	RHBDD2	2.399513	RRN3	1.720919	SETD2	1.711178
PUSL1	1.826275	RHBDD3	2.344917	RRP1	1.936495	SETD8	1.544776
PVRL4	2.720911	RHBDL3	0.627178	RRP12	2.91339	SETX	2.087393
PYCR2	1.321876	RHOT2	1.856525	RRP7A	1.496766	SF4	1.525127
PYGB	1.635777	RIC8B	1.381082	RSC1A1	1.649713	SFRP1	0.707334
QARS	0.751097	RLF	1.568608	RSHL3	2.564781	SFRS12	1.547123
QPRT	0.740909	RN5S9	2.381664	RSPH3	1.348531	SFRS15	1.371187
QTRT1	1.649737	RNF10	1.499589	RSRC2	1.899226	SFRS16	2.568433
RAB10	0.638302	RNF121	1.385695	RTN4IP1	0.643549	SFRS17A	1.416167
RAB11A	0.71329	RNF145	0.449837	RUFY1	1.787593	SFRS4	1.330607
RAB36	1.442233	RNF169	1.933091	RUSC2	1.960358	SFRS8	1.734089
RAB6B	0.603801	RNF25	3.063573	S100A4	0.339569	SFXN1	0.661014
RAB7L1	0.540018	RNF5P1	0.527336	SAC3D1	1.343258	SGK1	0.611934
RAB9B	0.602144	RNMT	0.725566	SAFB	1.386667	SH2B3	0.707156
RAD23B	0.610291	RNU1-3	4.704136	SAFB2	1.887179	SH3BP5L	2.390083
RAD9A	1.731587	RNU1-5	3.910265	SAMD5	0.642081	SHARPIN	1.814249
RAGE	1.927727	RNU1A3	3.14427	SAMD9	0.611498	SIAH2	0.573507
RALA	0.712642	RNU1F1	2.603112	SAP18	0.569948	SIGMAR1	1.447468
RALB	0.755952	RNU1G2	4.704983	SAPS1	1.413285	SIRT1	1.338698
RALGAPB	1.649335	RNU6-1	2.012903	SAT2	1.320696	SIX1	0.68283
RAP2A	0.628372	RNU6-15	2.030369	SBDS	3.281936	SIX5	0.705349
RAPGEF6	1.690295	RNU6ATAC	3.434213	SCAMP2	1.95679	SKAP2	0.512355
RASSF1	3.431152	RNU86	5.522851	SCHIP1	0.703505	SLC12A4	2.318422
RASSF7	1.309394	RNY1	2.335576	SCOC	0.596394	SLC19A1	1.404782
RBBP9	0.510045	RNY4	9.847384	SCYL1	1.231546	SLC1A3	1.816631
RBKS	2.384219	RP11-529I10.4	1.635376	SDCCAG8	1.53268	SLC22A15	1.769267
RBL2	0.600561	RP9P	1.892414	SDHAP2	1.569973	SLC30A7	1.709059
RBM15	1.469374	RPA4	2.2545	SDHD	0.698239	SLC35A5	0.522042
RBM17	0.714041	RPAP1	2.72895	SEC22A	1.273703	SLC35B4	0.710933
RBM38	1.663764	RPF2	1.254763	SEC62	0.665171	SLC35D3	0.432721
RBM42	2.007018	RPL13A	0.614286	SELENBP1	0.689808	SLC36A4	1.932892
RBM6	1.334168	RPL15	0.619332	SELM	1.330965	SLC37A4	0.72596
RBMS2	1.441779	RPL22	0.460995	SELO	1.555869	SLC38A2	0.625865

GENE	FOLD CHANGE	GENE	FOLD CHANGE	GENE	FOLD CHANGE	GENE	FOLD CHANGE
RBMX	0.707762	RPL7A	0.764916	SEMA3A	0.349381	SLC3A2	2.286919
RC3H2	1.80394	RPN2	1.580817	SEMA6A	0.653214	SLC44A1	0.756781
RDH13	1.93029	RPS12	0.711977	SEPW1	1.422866	SLC47A1	0.592914
RECQL4	1.762142	RPS23	0.664122	SERPINB1	0.526028	SLCO4C1	0.533323
REEP5	0.693837	RPS26L	1.347589	SERPINE2	0.71716	SLIT2	0.568608
RFWD3	1.648038	RPS6KA4	1.585228	SERTAD1	2.665969	SLITRK5	0.477888
RFX7	0.690276	RPUSD4	0.725226	SETD1A	1.338584	SMPD2	2.030161
SMS	0.665245	ST6GALNAC6	1.603308	TMED4	0.686012	TRMT61A	3.367321
SMYD4	1.82649	STAG1	1.445502	TMED7	0.583515	TRMU	2.445397
SNAPC4	1.746439	STK11IP	1.995264	TMEFF2	0.611426	TRNAU1AP	0.524434
SNCA	0.456328	STK35	1.706123	TMEM101	1.332996	TSHZ1	0.44864
SNHG12	5.90251	STK36	1.479917	TMEM11	1.418095	TSHZ3	0.507443
SNHG6	1.60706	STRADB	0.678091	TMEM117	0.666677	TSPAN3	0.647955
SNORA10	1.449241	STRN3	0.609675	TMEM123	0.640744	TSPAN5	0.616689
SNORA76	0.621701	STS-1	0.635589	TMEM194	1.292186	TSPAN7	0.56403
SNORD13	2.32216	STX1A	3.205996	TMEM198	3.041822	TSR2	1.463732
SNORD14A	0.606824	STX3	1.313373	TMEM206	0.684805	TSSC4	1.439874
SNORD31	0.475899	SUCLA2	0.550842	TMEM214	1.786968	TSTA3	1.931261
SNORD3A	2.284414	SUCLG2	0.716217	TMEM222	1.800096	TTC25	1.852716
SNORD3C	3.054952	SUMO2	0.459057	TMEM35	0.549476	TTC31	0.659301
SNORD3D	3.70108	SUMO3	0.66268	TMEM41B	0.777819	TTC32	0.701464
SNORD55	2.554099	SUPT5H	1.720498	TMEM52	0.498973	TTC38	1.580782
SNTB1	0.710682	SUPT6H	1.787185	TMEM59	0.751992	TTLL5	1.429938
SNUPN	0.591322	SURF2	1.73562	TMEM62	1.733836	TTLL7	3.074307
SNX4	0.64535	SURF6	1.473829	TMEM90B	0.364559	TUBB	0.62128
SOX2	0.527592	SUSD3	0.471205	TMSB15A	0.513611	TUBB2B	0.749189
SOX9	0.677152	TAF10	1.409894	TMX3	0.590393	TUFT1	1.645002
SPAG7	0.654007	TAF5	0.74793	TNFAIP3	9.464084	TUSC2	1.6215
SPATA2L	1.932527	TAF9L	0.540435	TNFRSF12A	2.461582	TWIST1	0.755652
SPINK2	0.424122	TAP1	1.300362	TNIP1	1.537438	TXNDC15	0.599193
SPINK5	2.084763	TAPT1	1.874492	TNPO2	1.375042	TXNL4B	2.543607
SPON1	0.594311	TATDN2	1.417321	TOM1L1	0.526343	TYW1B	1.645455
SPRY1	0.552863	TBC1D22B	1.789916	TOP3B	1.630439	U2AF1L2	1.867539
SPRYD4	0.541042	TBL3	1.847235	TOPBP1	1.483314	UAP1L1	2.93623
SPTLC2	0.527134	TDG	1.340274	TOR1AIP2	1.896146	UBA6	1.47313
SQSTM1	1.684961	TEX15	0.661472	TP53BP1	1.443503	UBAP2	1.917633
SRI	0.560342	TFPT	1.533055	TPX2	1.279272	UBE2J1	0.724342
SRM	1.811383	TGFBR3	0.742276	TRAFD1	1.928192	UBE2N	0.547408
SRPX	0.437762	THADA	2.109544	TRIB1	0.585286	UBL3	0.680444
SRRM1	1.31728	THEM2	0.727146	TRIM11	1.481113	UBQLN4	1.465548
SSBP2	0.653388	THOP1	2.028412	TRIM26	1.953049	UBR2	1.920508
SSH2	1.643538	THUMPD2	2.012158	TRIM28	1.420569	UBR5	1.651336
SSR1	0.664453	TIMM44	1.550721	TRIM45	0.610226	UCRC	0.558274
SSX2IP	0.617741	TIMP3	0.582857	TRIM47	2.330327	UFSP1	1.672673
ST13	0.768317	TJAP1	1.315854	TRIP11	1.709565	UIMC1	1.628987
ST3GAL2	0.730287	TLE1	0.736168	TRIT1	2.861232	UMPS	0.377504

GENE	FOLD CHANGE	GENE	FOLD CHANGE	GENE	FOLD CHANGE	GENE	FOLD CHANGE
ST3GAL6	0.658982	TLE4	0.562466	TRMT1	1.471494	UNC84A	0.690427
UPLP	1.260867	ZFAND2A	1.618728	ZNF57	2.604265	USP39	1.662061
USO1	0.62023	ZFAND3	1.99262	ZNF579	1.523326	USP5	1.286221
UST	0.685135	ZMAT3	2.009283	ZNF707	1.583152	XPC	1.394685
UVRAG	1.683306	ZMIZ1	0.694675	ZNF721	2.229187	XRCC1	1.413745
VASN	1.650987	ZNF140	2.046358	ZNF764	2.158272	XRCC3	1.792883
VGF	2.887397	ZNF148	1.31221	ZNF767	2.457453	XRCC5	0.593159
VPS18	1.741878	ZNF165	1.567898	ZNF773	2.105024	YEATS4	0.529247
VPS33A	0.567015	ZNF202	1.730452	ZNF777	1.421824	ZADH2	0.65514
VPS33B	1.493006	ZNF205	1.697957	ZNF783	1.785127	ZBTB17	2.120927
VPS37B	1.884272	ZNF211	1.738662	ZNF787	1.64261	ZBTB22	0.579712
WARS2	0.63884	ZNF213	1.586769	ZNF79	4.006687	ZBTB42	0.601997
WASF2	1.747912	ZNF25	0.392197	ZNF791	1.883901	ZBTB43	1.844487
WASH2P	2.074583	ZNF250	1.834842	ZNF821	1.305265	ZBTB46	2.16424
WBSCR22	1.448464	ZNF256	1.792453	ZNF845	1.882445	ZBTB48	1.758466
WDR25	2.33665	ZNF26	1.351611	ZNHIT2	1.90252	ZBTB5	0.671512
WDR41	0.606265	ZNF277	0.691786	ZNRF3	0.545048	ZC3H18	1.7459
WDR55	1.532463	ZNF282	1.687195	ZP3	2.119617	ZC3H3	1.52804
WDR60	1.540387	ZNF296	2.018435	ZRSR2	2.87585	ZDHHC18	1.475063
WDR62	2.362981	ZNF304	1.676241	ZSCAN12	2.493271	ZFP36	1.558652
WDR79	1.36084	ZNF317	1.545945	ZSCAN21	1.329037	ZFP82	1.534037
WHAMM	2.659794	ZNF32	0.513408	ZZZ3	1.523812	ZNF343	1.504112
ZNF35	1.378232	WNT5A	0.44706	WNT5A	0.44706	ZNF416	1.595944
ZFPM1	1.548313	ZNF364	1.370786	ZNF364	1.370786	ZNF419	1.546236
ZNF324	2.175526	ZNF383	1.988769	ZNF383	1.988769	ZNF426	1.524803
ZNF431	2.044255	ZNF433	2.042966	ZNF444	1.595615	ZNF446	1.42368
ZNF470	1.87809	ZNF480	1.542453	ZNF500	1.432364	ZNF557	1.5631
ZNF598	1.326032	ZNF627	1.443742	ZNF630	1.601751	ZNF696	1.571802

Supplementary Table S3: The list of 428 differentially expressed genes after depletion of hsa-miR-195 in MCF7 cells

GENE	FOLD CHANGE	GENE	FOLD CHANGE	GENE	FOLD CHANGE	GENE	FOLD CHANGE
AEN	1.677893	C20ORF111	1.337153	CXORF39	2.337974	GTF3C5	1.358446
AKR1C3	0.690353	C20ORF52	1.384663	CYP27B1	0.689505	HAVCR2	0.330279
ALPL	0.621651	C21ORF122	0.51669	CYP4V2	0.585563	HESX1	0.386351
ANGEL1	1.461615	C4ORF49	0.607578	DAPK3	2.019278	HIST1H2AC	0.494899
ANKRD1	9.156219	C6ORF129	1.313886	DCP2	1.559785	HIST1H4K	2.090867
ANKRD54	1.487245	C6ORF130	0.734589	DDB2	1.662713	HIST3H2A	0.690726
APBA3	1.374231	C6ORF160	1.50088	DDX39	1.211225	HJURP	1.441844
ARC	1.816252	C6ORF173	1.60675	DKFZP686K1684	0.636709	HLA-E	1.433855
ARF3	1.582442	C7ORF40	1.82021	DNTTIP1	1.297843	HSD17B6	0.614904
ARMC1	1.32901	C7ORF47	1.312705	DPP9	1.380227	HSPB8	1.709626
ASGR1	0.586126	C8ORF59	1.613759	DUS3L	1.463481	HTRA1	0.698507
ATAD2	1.428275	CBR4	0.676108	DUSP5	1.449689	IER3	2.115312
ATAD3A	1.487463	CCDC110	0.526746	DYNLL2	1.389282	IGSF5	0.412783
ATL3	1.435411	CCDC72	1.378639	E2F5	0.679776	IL32	5.49949
ATP5G1	1.54823	CCDC86	1.433949	ECT2	1.568842	IL8	5.982008
ATP5J2	1.348867	CCDC92	0.659714	EDC3	1.52483	IRF1	1.53213
AURKAIP1	1.346425	CCL2	2.080626	EIF2S1	1.443871	ISG20L1	1.740436
AVL9	1.581566	CCT5	0.607402	EIF3A	1.691591	KIAA0586	1.49218
AYP1P1	1.374888	CD70	1.582662	ELP4	0.675574	KIAA1731	1.99825
BCL3	2.171031	CDC25A	1.370662	ERF	1.343913	KIF2C	1.469618
BLOC1S2	1.611136	CDCA2	1.548794	ERRFI1	0.7342	KIF4A	1.309953
BRI3	1.340152	CDCA3	1.610177	FAM133B	1.34364	KIFC1	1.366926
BRI3P1	1.546793	CDCA5	1.336007	FAM158A	2.238459	KRT222	0.613685
BTN3A3	1.95368	CENPBD1	0.681338	FAM3A	1.361278	LACTB2	0.621562
BUB1	1.29454	CENPE	1.215363	FAM46C	1.520561	LAGE3	1.336476
BUB1B	1.777002	CENPN	1.286913	FAM98A	1.868986	LENG1	1.663116
C10ORF137	1.4131	CENPQ	2.055474	FAU	1.301277	LGALS1	1.543264
C10ORF41	0.502603	CHD1	1.374563	FDPS	1.374176	LINS1	1.514225
C11ORF70	0.547787	CHERP	1.374179	FDX1L	1.457109	LOC100128731	1.353418
C12ORF26	0.646565	CHMP1A	1.367303	FHOD1	1.335856	LOC100128936	1.474862
C12ORF45	1.331408	CHP2	0.449325	FLJ22795	1.649252	LOC100129028	2.428756
C14ORF156	1.429685	CIR1	1.65824	FLJ43681	1.830567	LOC100129297	1.656124
C14ORF93	0.71006	CKS2	1.647693	FRG1	1.610092	LOC100129379	1.752259
C15ORF38	0.64098	CMC1	1.331083	GADD45A	1.396641	LOC100129650	1.441238
C15ORF42	1.547957	CORO7	1.358894	GMPR	0.660258	LOC100130003	1.454882
C16ORF52	0.618152	COX6B1	1.378484	GNG4	1.45125	LOC100130070	1.724371
C19ORF22	1.377093	COX7B	1.717472	GNGT1	1.858629	LOC100130154	2.226066
C19ORF44	0.648019	CPOX	1.692286	GPATCH3	1.620661	LOC100130886	0.69605
C1ORF35	1.33793	CXCR7	0.5446	GPR172A	1.348516	LOC100131785	1.335019
LOC100131801	1.3194	LOC645726	1.919625	MGC71993	1.281378	ODF2	1.276451
LOC100132037	1.442311	LOC645937	1.411599	MGC87895	1.509317	ORC1L	1.518546
LOC100132418	1.42454	LOC646197	1.617086	MICB	1.371286	OSAP	0.661231
LOC100132457	1.89355	LOC646576	0.500334	MIR221	1.357492	PATL1	1.38577
LOC100132499	1.60235	LOC646753	1.46071	MIR886	2.361266	PELP1	1.61311
LOC100132948	1.68382	LOC646819	1.500187	MKL2	2.551981	PFAS	1.380042
LOC100133390	1.500664	LOC647037	1.811877	MKX	0.712526	PHF21B	1.718663
LOC100133812	1.365288	LOC648622	1.633054	MPHOSPH6	1.609677	PHLDA3	1.983609
LOC100133887	0.362622	LOC648638	1.900106	MRPL10	1.631106	PHPT1	1.43751
LOC100134537	1.659417	LOC649548	1.401815	MRPL15	1.430061	PIBF1	1.525063
LOC144438	1.559465	LOC649839	2.078199	MRPL19	1.346894	PISD	1.432158
LOC151579	1.396555	LOC650298	1.567115	MRPL41	1.371887	PLA2G4C	2.367569
LOC284988	1.883653	LOC652545	1.540334	MRPS17	1.418166	PMS2L3	1.478905
LOC338870	1.835081	LOC652672	1.33077	MT1A	2.523131	PNMA2	0.693021
LOC347292	1.603262	LOC653874	1.714328	MT1F	1.485696	POMT2	0.687886
LOC347376	3.224295	LOC654121	1.431988	MT1X	2.533954	PPP1R12C	1.903344
LOC387791	1.471175	LOC727758	0.614924	MT2A	2.779558	PRMT10	1.386457
LOC388339	1.547226	LOC727865	1.251827	MTE	3.159964	PTTG3P	1.49936

GENE	FOLD CHANGE	GENE	FOLD CHANGE	GENE	FOLD CHANGE	GENE	FOLD CHANGE
LOC391019	1.571402	LOC728006	0.681138	MTIF3	0.707748	RALGAPB	1.417705
LOC391126	1.504997	LOC728244	1.940155	MTMR6	1.607461	RASSF1	2.065798
LOC391370	1.874656	LOC728590	1.891131	MVD	1.423771	RBM17	0.646229
LOC391656	1.592894	LOC728823	2.01042	MYBBP1A	1.504475	RBM26	1.553303
LOC392437	1.367284	LOC729362	1.339078	MYBL2	1.727078	RDH13	1.422977
LOC399491	1.524466	LOC729421	0.544916	NADSYN1	1.345947	RECQL4	1.397307
LOC400948	1.391886	LOC729608	1.303723	NCAPD2	1.456334	RFWD3	1.414772
LOC440145	1.927142	LOC729646	1.903514	NCAPD3	1.325066	RHOT2	1.532961
LOC440354	1.970208	LOC729769	1.574934	NCAPG	1.33953	RIOK3	1.546605
LOC440957	1.519554	LOC729816	1.327598	NDC80	2.020453	RN5S9	1.417495
LOC440991	2.059077	LOC730183	0.64682	NDUFA3	1.366374	RNF181	1.291151
LOC441377	1.850447	LOC731640	2.144683	NDUFB10	1.349643	RNU1-3	2.464397
LOC442454	1.761064	LOC731985	1.46807	NDUFB9	1.689956	RNU1-5	1.759004
LOC641768	2.267177	LOC92755	1.254673	NEIL2	2.501403	RNU1A3	1.804236
LOC642033	1.604119	LSMD1	1.377138	NEK2	1.594701	RNU1F1	2.45227
LOC643358	1.478226	LTBP2	0.379304	NEU1	1.547135	RNU1G2	2.201603
LOC643949	1.572175	MAP2K2	1.391497	NFKB1	1.415937	RNU4ATAC	2.002771
LOC644033	1.338065	MAP3K8	2.078596	NFKBIA	1.396611	RNU6-1	1.600701
LOC644928	1.443024	MAP7D2	0.723802	NFKBIB	1.926538	RNU6-15	1.606429
LOC645233	2.069008	MCM5	1.571462	NIF3L1	0.717231	RNU86	3.12876
LOC645387	1.2515	METRN	1.710783	NPM3	1.349797	RNY4	4.165886
LOC645586	1.798766	METTL11A	1.665658	NRAS	1.462199	ROMO1	1.532933
RP9P	1.564468	SHROOM3	1.459797	STX8	1.294574	TTLL7	1.93222
RPL12P6	1.725901	SKIV2L	1.429712	SUGT1	0.680271	TUBD1	1.759565
RPL23AP7	1.348205	SLC2A3	1.277281	SYT11	0.716484	TUT1	1.605766
RPN2	1.891316	SLC35A5	0.645529	TACC3	1.451563	TXNDC17	1.437818
RPRML	0.689465	SLC35B3	0.675568	TAF10	1.46912	UBA6	1.708582
RPS15	1.272556	SLC36A4	1.586172	TAP1	1.293056	UBAP2	1.671385
RPS21	1.392038	SLC5A6	1.321955	TBL3	1.638652	UBL5	1.395871
RPS23	0.734562	SLU7	1.321717	THAP10	0.612749	UBLCP1	1.426508
RPS26L	1.422297	SMNDC1	1.424433	THOP1	1.349524	UHRF2	1.450225
RPS28	2.480285	SNAP29	0.703016	TIMM8A	1.576699	USP30	1.416457
RRP1	2.029517	SNHG12	2.740855	TMEM166	0.659475	UST	0.717473
RRP12	1.827857	SNHG6	1.442716	TNFAIP3	10.11115	UTP14A	1.472158
RRP7A	1.330567	SNORA10	1.362984	TNFRSF9	5.854754	VGF	1.699903
RSRC2	1.36793	SNORA59B	1.902207	TNIP1	1.35603	VPS33B	1.324278
SAP18	0.468024	SNORD3A	1.510705	TOMM20L	0.365857	VPS4A	1.411978
SBDS	2.143475	SNORD3D	1.8649	TOMM5	1.438556	VTI1B	1.459415
SCNM1	1.369459	SNORD55	1.879463	TPX2	1.345564	WDR34	1.298855
SDC4	1.478484	SNORD56	1.461978	TRAF2	1.530028	YES1	1.338852
SDHD	1.344804	SNRPF	1.328464	TRIM26	1.32933	ZADH2	0.634956
SEC61B	1.370921	SNRPG	1.357171	TRIP12	1.777271	ZNF252	0.716161
SELM	1.403241	SPATA5L1	1.649718	TRIT1	1.660886	ZNF304	1.697754
SEPW1	1.501067	SPG3A	1.463355	TRMT61A	1.731303	ZNF341	2.266905
SERTAD1	1.612934	SQSTM1	1.446502	TSHZ1	0.622391	ZNF431	1.926174
SH3BP1	1.666441	SRM	1.31773	TSHZ3	0.62652	ZNF467	0.668831
SHFM1	1.365765	STIL	1.537199	TTK	1.355852	ZNF583	1.326019
ZNF764	1.761085	ZNF787	1.341326	ZNF845	1.506964		

Supplementary Table S4: List of differentially expressed genes showing significant fold difference of expression levels in case of hsa-miR-195 overexpression and inhibition in MCF7 cells

Gene name	Fold change after miR-195 overexpression	Fold change after miR-195 inhibition	Difference in Fold change
PLA2G4C	9.91	2.368	4.186
PHF21B	4.535	1.719	2.639
RNY4	9.847	4.166	2.364
RNU1-5	3.91	1.759	2.223
SNHG12	5.903	2.741	2.154
RNU1G2	4.705	2.202	2.137
SNORD3D	3.701	1.865	1.985
GNGT1	3.632	1.859	1.954
TRMT61A	3.367	1.731	1.945
RNU1-3	4.704	2.464	1.909
LOC440354	3.742	1.97	1.899
ARC	3.396	1.816	1.87
ANKRD54	2.725	1.487	1.832
RNU86	5.523	3.129	1.765
AEN	2.927	1.678	1.744
RNU1A3	3.144	1.804	1.743
TRIT1	2.861	1.661	1.723
HIST1H4K	3.566	2.091	1.705
CCDC86	2.442	1.434	1.703
VGF	2.887	1.7	1.699
RN5S9	2.382	1.417	1.68
RASSF1	3.431	2.066	1.661
SERTAD1	2.666	1.613	1.653
PMS2L3	2.409	1.479	1.629
DAPK3	3.233	2.019	1.601
RRP12	2.913	1.828	1.594
TTLL7	3.074	1.932	1.591
DUSP5	2.287	1.45	1.578
KIAA1731	3.079	1.998	1.541
SBDS	3.282	2.143	1.531
DUS3L	2.215	1.463	1.513
SNORD3A	2.284	1.511	1.512
THOP1	2.028	1.35	1.503
C10ORF41	0.318	0.503	-1.58
LOC100129028	1.484	2.429	-1.64
HSD17B6	0.365	0.615	-1.68
SDHD	0.698	1.345	-1.93

Supplementary Table S5: The list of networks generated during Ingenuity pathway analysis using the differentially expressed genes obtained after hsa-miR-195 over-expression in MCF7 cells

Molecules in Network	Score	Focus molecules	Top functions
ACIN1, AKR1C3, Akt, AKT1S1, ANGEL1, ARL1, ARMC6, ATP5L, CADM1, CHFR, CREG1, DNA-directed RNA polymerase, EFTUD2, FOXC1, INPP5E, MIRLET7D (includes EG:406886), MPP3, NEXN, NOC2L, PDXDC1, PHLDA3, POLR3A, POLR3D, PROS1, QARS, RNF115, RPAP1, RPN2, SAP18, SFXN1, SLC3A2, STK36, TGFBR3, ZC3H18, ZZZ3	46	33	Cellular Movement, Reproductive System Development and Function, Cardiovascular Disease
BTF3L4, CTDP1, DDX56, DRAP1, ERF, FANCI, GAS1, GNL2, GTF2F1, HELZ, HLA-DR, HLA-DRA, HLA-DRB1, Holo RNA polymerase II, MRPS5, MYBBP1A, MYC, MYCBP2, NELF, PAF1, POLR2A, RAGE, RNA polymerase II, RPL15, RRP12, SAFB, SETD2, SLC27A2, SPAG7, SSBP2, SUPT5H, SUPT6H, TAF10, TAF9B, UST	44	32	Gene Expression, RNA Post-Transcriptional Modification, Antigen Presentation
ATL2, AUH, AUTS2 (includes EG:26053), C12ORF44, CCND2, COPA, CTSC, DKK3, ECH1, enoyl-CoA hydratase, ERP29, EWSR1, FGFBP1, GBP2, HADHA, HADHB, IgG, Immunoglobulin, Interferon alpha, MED4, MED14, Mediator, NKTR, NLE1, PAFAH1B3, PATL1, PDIA6, PRKAB1, RSRC2, SERTAD1, TRIM28, UBQLN4, WBSCR22, ZNF205, ZNRF3	39	30	Genetic Disorder, Metabolic Disease, Lipid Metabolism
AATF, BBS2, C15ORF23, C5ORF13, COMMD1 (includes EG:150684), CORO1A, DCP2, DCP1A, E2F7, EDC3, GNGT1, GPC4, IRAK2, LITAF, MHC CLASS I (family), MPP6, MRPL38, NFKB1, NFkB (complex), NFkB (family), NFKBIB, NOP14, Pro-inflammatory Cytokine, Serine Protease, SIAH2, SQSTM1, SUMO3, THOP1, TNFRSF12A, TNIP1, TRAFD1, TUBB2B, Ubiquitin, ZFP36, ZNF202	36	29	Hematological Disease, Organismal Injury and Abnormalities, Gene Expression
ACTA1, Alp, ALPL, APBA3, APP, ARL6IP5, BCKDK, BIRC6, C14ORF147, Caspase, CBR4, DNAH1, DUSP14, FSH, GCA, GPS2, HSD17B8, IARS2, IGFBP5, JUNB, KDM5B, Lh, LTBP2, PBK, PPAP2A, PPP2R5B, PSEN2, PSMD3, SMPD2, SPON1, SRI, SUCLA2, Tgf beta, VGF, ZNF773	35	30	Lipid Metabolism, Molecular Transport, Small Molecule Biochemistry
AP1S2, Ap2 alpha, ATPase, C3ORF1, CBL, CDK20, DLX1, DUSP5, EPHA4, GHR, GPC3, growth factor receptor, HGS, IER5L, ILKAP, INO80, INO80D, IPO13, NAPB, p85 (pik3r), PAX6, Pdgf, PDGF BB, PDGFD, PDX1 (includes EG:3651), PELP1, PICK1, Pkc(s), PLC gamma, PPP1R15A, SOX2, TIMP3, TRIM11, VPS37B, WNT5A	33	27	Cellular Development, Cellular Growth and Proliferation, Cell Morphology
ADAP2, ANKRD1, CHD8, ENaC, ERK1/2, FKBP11, GRN, HERP, HEY1, HEY2, HIST1H1C, KLF13, METAP2, Notch, P-TEFb, peptidase, peptidylprolyl isomerase, PPI, PPIA (includes EG:5478), PPIC, PRDX6, PRSS8, RNF10, RNU1-4, S100A4, Secretase gamma, SEMA3A, SERPINE2, SH2B3, SLC12A4, SNAPC4, SNCA, TOP3B, VASN, ZFPM1 (includes EG:161882)	31	26	Cellular Growth and Proliferation, Nervous System Development and Function, Tissue Development
APC, CCDC76, CCNB1, CDC5L, Cyclin A, Cyclin B, Cyclin E, DAPK3, DAXX, E2f, E2F5, E4F1, Hdac, ID2, KIAA0240, LIN37, MAP1S, MCM2, MCM5, MDM4, NOP2, PCSK7, PCSK1N, PEA15, PRPF19, Ras, RASSF1, Rb, RBBP9, RBL2, RBMX, TSHZ3, Xrcc, XRCC1, XRCC3 (includes EG:7517)	30	26	Cell Cycle, DNA Replication, Recombination, and Repair, Connective Tissue Development and Function
ACTR6, ADH5 (includes EG:128), Ahr-aryl hydrocarbon-Arnt, alcohol dehydrogenase, BCL3, BCL11A, COL13A1, Ctbp, CYP1B1, CYP27B1, CYP3A5, DHRS2 (includes EG:10202), DUSP8, EDA2R, EPC1, ERRFI1, glutathione transferase, GST, GSTA1, GSTA4, GSTM2, ISL1, Jnk, NR1H3, NR2F1, PECR, PIR, PXR ligand-PXR-Retinoic acid-RXRα, RAB7L1, Rxr, SERPINB1, TIP60, TRIP11, VitaminD3-VDR-RXR, YEATS4	28	25	Drug Metabolism, Glutathione Depletion In Liver, Lipid Metabolism
ADAMTS1, ASB9, BNIP3L, C1ORF182, CCT4, COL5A1, collagen, Collagen(s), Cpla2, CTSL1, EXOC3, F12, FAM40A, FTH1, GADD45B, GADD45G, Hsp27, Hsp70, HSPB8, IL1, IL32, KLF9, NCOA5, P38 MAPK, PLA2, PLA2G7, PLA2G4C, Pld, PLSCR3, RALA, RAP2A, RAPGEF6, RPS6KA4, Sapk, STRN3	27	25	Cell Signaling, Carbohydrate Metabolism, Lipid Metabolism
ASCC2, CD19, EEA1, ELAC2, Gamma tubulin, HMOX1, IFIT2, IFN Beta, IL12 (complex), IL28RA, IRG, ISG15, JAK, KDM3A, KDM5A, KIT, LDL, LIPA, LT, LTA, LTBR, MKI67, NAPG, PI3K (complex), PIK3R4, PON2, Rab5, RUFY1, SLIT2, STAT5a/b, TNFAIP3, TRAF, UVRAG, VAV, VPS18	25	23	Cardiovascular System Development and Function, Lymphoid Tissue Structure and Development, Organismal Development
ATRIP, Cbp/p300, Cofilin, DDB2, DPYSL2, ERCC2, ERCC3, ERK, GRWD1, Importin beta, IPO8, KPNA1, KPNA4, POLE, RAD23B, RAD9A, RNA polymerase I, Rock, RPA, RPA4, RRN3, SAA, Smad2/3-Smad4, SNUPN, SOX9, TDG, TFIIA, TFIIF,	25	23	DNA Replication, Recombination, and Repair, Nucleic Acid Metabolism, Small Molecule Biochemistry

Molecules in Network	Score	Focus molecules	Top functions
TFIIH, TOPBP1, TP53BP1, UBR2, UBR5, XPC, ZBTB17			
ARPC1B, ATL3, C11ORF75, C16ORF48, C6ORF72, DHX8, DMWD, DPPA2, DVL3, DVL2 (includes EG:1856), ENDOD1, FXR2, MAPK1IP1L, MIR124, MOBKL2B, NOC3L, NT5C2, PLOD3, PRPF3, RAD51AP1, SDF4, SLC30A7, SRM, SUCLG2, TCEB3B, THAP1, TRIM29, TSR2, USP5, USP39, ZBTB8A, ZNF165, ZNF250, ZNF263, ZNF446	25	23	RNA Post-Transcriptional Modification, DNA Replication, Recombination, and Repair, Metabolic Disease
Ap1, ARL2, Arp2/3, ASGR1, Calmodulin, CaMKII, CEP250, Ck2, Creb, CSPG5, EIF3, EIF2A, EIF3B, EIF3D, EIF3J, EIF4G3, F Actin, GAD1, MGMT, MICALL1, MYH3, MYL5, Myosin, PACSIN1, PIP4K2A, PLS1, POLM, PP1 protein complex group, PP1-C, PPP1CB, PPP1R14C, SIX1, SSR1, Tubulin, XRCC5	24	23	Protein Synthesis, DNA Replication, Recombination, and Repair, Connective Tissue Disorders
ACVRL1, ADAM12, AP3S1, BUD31 (includes EG:8896), C6ORF130, CNTNAP2, CTR9, DFNA5, E2F5, EEA1, EGLN1, ENO1, G3BP1, HSD17B6, KDM6B, KRI1, LDB1, NKX3-2, NOL6, NUP214, PAPPA, PDLIM5, PIAS3, PIGF, RUNX3, SMAD2, SNIP1, SSBP4, SURF6, TGFB1, TGFBR3, TLE4, TPM3, VASN, ZMIZ1	22	21	Gene Expression, Cellular Development, Cellular Growth and Proliferation
ADCY, ADORA2B, CELSR3, CXCR7, DOCK2, Focal adhesion kinase, FZD4, G protein, GABBR2, GALR2, Gpcr, GPER, GPR37, GPR64, GPR160, GPR172A, Gs-coupled receptor, Gsk3, HBEGF, IL12 (family), LPAR3, LPXN, Mmp, NPY, Pias, PIAS3, Pka, PLC, PTGER1, PTGER2, Rac, SRC, STAT, ZNF426, ZP3	22	21	Cancer, Neurological Disease, Cardiovascular System Development and Function
ACBD6, C11ORF54, CCDC45, CHERP, GMDS, HNF1A, HNF4A, ISOC1, LSM3, MCCC1, RBKS, RSPH3, RTN4IP1, SFRS8, SMAD3, TTC25, TUFT1, TXNL4B, WARS2, WBP4, ZNF277	19	16	Gene Expression, Embryonic Development, Tissue Development
ARRB1, ATAD3A, BAT1, BAT5, BCLAF1, BUD13, CCDC93, CCL14, CHCHD3, CHD1 (includes EG:1105), CWC22, DDX39, DHRS2 (includes EG:10202), DHX16, FRMD4A, GPKOW, GRK6, HNRNPA1, HNRNPA1L2, IGF2BP2, KIN, LARP7 (includes EG:51574), LMNB2, MRPS27, PIP, PPIA (includes EG:5478), PRSS23, RBMX2, RECQL4, SAFB, STOM, THOC4, TMEM222, TRAF3IP1, ZBTB43	19	19	RNA Post-Transcriptional Modification, Molecular Transport, RNA Trafficking
AHSA2 (includes EG:130872), APPBP2, ARID3B, BCL3, BDP1, BNC2, BRF1, CMAS, CREG1, DNAH1, DNASE1, F2R, Gcn5l, HSP90AA1, IKZF1, IRF1, KDM5A, MDM4, MED4, NR3C1, PI4KAP2, RB1, RBMS2, RNASEN, SNAPC1, SNAPC3, SPTAN1, STRADB, TAF4B, TBL3, TBP, TNFAIP3, TRIM45, TSSC4, WDR62	18	19	Gene Expression, Respiratory Disease, Digestive System Development and Function
C19ORF42, FAM158A, HIST1H4K (includes EG:8362), HMGN4 (includes EG:10473), HNF4A, KLHL28, LRIG2, NUDT11, ONECUT1, SDR39U1, SLC35A5, TMEM59 (includes EG:9528), TRAF6, TRMT6, TRMT61A, TTC35, TTC38, ZNF304, ZNF324, ZNF557	18	15	Digestive System Development and Function, Hepatic System Development and Function, Organ Development
AKAP1, C16ORF74, C5ORF41, CDKN1A, CIZ1, DAXX, DPF1, DPP3, FEN1, FGF13, GPS2, KBTBD8, LENG8, LGALS1, LIG1, MDM4, MIR106B (includes EG:406900), MIR17 (includes EG:406952), MIR196A2, MKX, NEUROG2, PARP2, PARP6, PLK2, PNPT1, PPP3CA, RB1CC1, RIMKLB, RNF121, SMOC2, SOX9, TP73, TTLL5, UBL3, XRCC1	17	18	DNA Replication, Recombination, and Repair, Cell Cycle, Cellular Development
CALM2, CCDC66, CENPT, CFL1, CLCN6, CTU2, DDX6, DHX29, FBXO33, FKBP8, GLCE, GNG4, KIAA1683, LRRC59, LSM12, MIR205 (includes EG:406988), MIR24-1 (includes EG:407012), MIR31 (includes EG:407035), MTCH2, MXI1, OFD1, PDGFRA, PHF21A, PISD, PRRX2, RAB2A, RAB5C, RAB9B, RIC8B, RNF10, RPL22, SDCCAG8, Tropomyosin, VHL, WDR55	17	18	Tissue Morphology, Carbohydrate Metabolism, Drug Metabolism
14-3-3, alcohol group acceptor phosphotransferase, BRAF, CAMK2N1, ECSIT, ETV5, HRAS, IKK (complex), Ikk (family), LGALS1, MAP2K2, MAP2K1/2, MAP3K, MAP3K1, MAP3K9, MAP3K11, Mapk, MAPK13, Mek, MUC16, Nfat (family), OIP5, Pak, PP1/PP2A, PRKCH, Raf, Rap1, RC3H2, RFWD3, RPS23, Sos, TCR, TRIB1, UBA6, UBE2N	16	20	Developmental Disorder, Genetic Disorder, Cellular Movement
CD79B, CDCA2, DAXX, DEXI, DUT (includes EG:1854), EDIL3, ELK3, ETS1, FAM3C, GOLM1, ING4, LMTK2, LTB, NEK2, NOC2L, P2RX4, PAFAH1B3, PPP1CA, PPP1CC, PPP1R2, PPP1R13B, PPP1R15A, PPP2R5C, RAD23B, REEP5, RPL13A, SCOC, SEC62, TAF1A, TMSB15A (includes EG:11013), TP53, ZAP70, ZMAT3, ZNF140, ZNF24 (includes EG:7572)	16	18	Cell-To-Cell Signaling and Interaction, Cellular Function and Maintenance, Cell Death
ADCK1, AIF1L, AP1G1, AP3S2, AP4E1, AP4M1, Arf, ARF1, ARF5, ARF6, ARFGAP1, ARFIP2, CAV2, CEP350, CLCN7, COPG, GBF1, GOLGB1, GOSR2, HOXA9, HOXB8, IRX3, IRX6, MIR210 (includes EG:406992), oleic acid, OSBPL8, PACS1, PBX3, PBXIP1, PIK3IP1, PLEKHM2, QTRT1, RAB1A,	16	17	Molecular Transport, Protein Trafficking, Cellular Assembly and Organization

Molecules in Network	Score	Focus molecules	Top functions
TMEM35, USO1			

Supplementary Table S6: The list of networks generated during Ingenuity pathway analysis using the differentially expressed genes obtained after hsa-miR-195 deletion in MCF7 cells

Molecules in Network	Score	Focus molecules	Top functions
ACLY, BUB1, BUB1B, CENPE, CKS1B, CSE1L, DHCR7, DUSP5, EIF2S1, EIF3A, GADD45A, IFN Beta, IFN TYPE 1, IgG, IRF1, IRG, ISG15, KRT8, LDL, MAD1L1, Mapk, NDC80, NEK2, PP1 protein complex group, PP1-C, PP2A, PPP1R1A, PRKRA, RPL5, RPS23, SLC2A3, STIL, TAP1, Vegf, YES1	38	25	Cell Cycle, Cellular Assembly and Organization, DNA Replication, Recombination, and Repair
ALPL, Ap1, CD70, COX6B1, COX6C, COX7B, COX8A, Cytochrome c oxidase, DNTTIP1, ELP4, Estrogen Receptor, FSH, GLRX2, Histone h4, Ifn gamma, Jnk, KIFC1, LSM7, LTBP2, MEPCE, MT1F, MYB (includes EG:4602), Pdgf, PELP1, PHLDA3, Pias, RANGAP1, Rxr, SNRPF, SNRPG, Tgf beta, TRIM26, TRIP12, TUT1, VGF	37	24	Energy Production, Cell Cycle, Cell Morphology
alcohol group acceptor phosphotransferase, Alpha tubulin, APC, AURKA, AURKAIP1, BIRC5, BTG2, CDC25A, CKS2, Cpla2, Cyclin A, Cyclin B, Cyclin E, CYP27B1, E2f, E2F5, ECT2, ERK1/2, Gamma tubulin, Hdac, IDE, KIF2C, MAP3K8, MAPKAPK5, MCM5, MT2A, MYBL2, ORC1L, PLA2G4C, RACGAP1, RASSF1, RPA3, TPX2, TSHZ3, TTK	36	24	Cell Cycle, Cellular Assembly and Organization, DNA Replication, Recombination, and Repair
26s Proteasome, Akt, ASGR1, BLOC1S2, Caspase, CCL2, CDCA3, ERRFI1, GNGT1, hCG, Hsp27, Hsp70, HSPA5, IFT57, Ikb, IL32, Importin beta, NfkB1-RelA, NFKBIA, NFKBIB, NIF3L1, PTTG1, RIOK3, SAA, Sapk, SDHA, SRP19, SSR4, STIP1, TRAF2, TRIAP1, TXNDC17, Ube3, Ubiquitin, USP5	32	22	Cancer, Hematological Disease, Organismal Injury and Abnormalities
BCL3, CAMK2N1, CD3, FTL, Gm-csf, HLA-E, IER3, IKK (complex), Ikk (family), LGALS1, MAP2K2, MAP3K, Mek, MHC Class I (complex), MHC CLASS I (family), MPHOSPH6, NFKB1, NFkB (family), NfkB-RelA, NRAS, PDGF BB, PEBP1, PI3K (complex), PIK3R4, Raf, RHOB, RPN2, SERTAD1, TCR, THOP1, TNFAIP3, TNFRSF9, TNIP1, TRAF, TYRO3	28	20	Cell Cycle, Gene Expression, Cell Signaling
ATAD3A, BTG2, CCDC92 (includes EG:80212), CDCA2, CDKN2AIP, CPOX, CTSK, EIF2S1, FGF2, HJURP, HNRNPA1L2, HTRA1, IFNB1, ISG15, MAD1L1, MKI67, MT1A, NEK2, NOC2L, PPP1CA, PPP1R10, PPP2R5C, PRKRA, RECQL4, RNU1-1, RPS28, S100A8, STOM, TACC3, TAP1, TERT, TOX4, TP53, TXNRD1, ZC3H4	27	20	Cellular Development, Cellular Growth and Proliferation, Nervous System Development and Function
ARF3, Calmodulin, CDCA5, CHMP1A, DCP2, EDC3, EDF1, ERK, F Actin, GDI1, GMPR, Histone h3, IL8, Immunoglobulin, KIAA0895, KIF23, MIR1, MSN, Nfat (family), NGF, P38 MAPK, PATL1, Pkc(s), Pld, PNMA2, Rac, RALA, Ras, Ras homolog, Rho gdi, RHOT2, SDC4, SH3BP1, SHROOM3, UST	24	20	DNA Replication, Recombination, and Repair, Cellular Assembly and Organization, Post-Translational Modification
AKR1C3, ANGEL1, CCHCR1, DEFB4A, EBF3, FABP6, FRG1, IFNGR2, ME1, MIRLET7D (includes EG:406886), MRPL19, MRPL41, MVD, NCF1C, NEU1, NFKB1, NKRF, NR4A2, PMS2L3, PTEN, RETN, SMURF2, T3-TR-RXR, TAL1, TARBP2, TICAM1, TNFAIP1, TNFAIP3, TNFRSF9, TP53BP1, TRAF, TRAF4, UTP14A, VPS4A	22	17	Immunological Disease, Gene Expression, Infectious Disease
CD3EAP, CKMT1A, GTF3C2, IKBKE, KIF11, LAGE3, METTL11A, MKX, MT-ND1, MT-ND4, NADH2 dehydrogenase, NADH2 dehydrogenase (ubiquinone), NDUFA1, NDUFA3, NDUFA6, NDUFA13, NDUFB7, NDUFB9, NDUFB10, NDUFS1, NDUFS8, NUTF2, PFAS, POLR3C, RNY4, SBDS, SDHA, SDHB, SDHD, SNORD3A, SSB, TBP, TNFAIP3, TROVE2, VEGFA	20	16	Cancer, Neurological Disease, Genetic Disorder
ANPEP, ARC, AUH, AZU1, BTN3A3, CD40LG, CSNK1D, DAPK3, DEFA1 (includes EG:1667), DYNLL2, EGF, Gelatinase, GNLY, HIST3H2A, IL15, IL32, IL1R2, Lh, MDM2, MICB, MIR152 (includes EG:406943), NFRKB, PAWR, POU2F1, RASAL2, RNF217, RNU6-1, RPS21, SLU7, THAP10, TNF, TNFRSF9, TNFSF9, TNIP1, VCL	20	16	Inflammatory Response, Drug Metabolism, Lipid Metabolism
ARL6, ASGR2, BAT3, C20ORF111, CDC45, CHERP, CIAO1, CIR1, COPB2, DPM1, GNL3, GTF3C5, HDAC8, HNF4A, LSMD1, MRPL15, MRPS15, MT1X, PHPT1, POLR1C, PSMB1, RPAP1, SEC61B, SHFM1,	19	15	Carbohydrate Metabolism, Nucleic Acid Metabolism, Small

Molecules in Network	Score	Focus molecules	Top functions
SLFN11, SNW1, SRP54, SRSF2, TROVE2, VIPAR, VPS41, VPS33B, WBP4, WBSCR22, ZNF764			Molecule Biochemistry
ANKRD1, Ck2, DDB2, DNAJC7, DNAJC10, Fibrinogen, GTF3C5, HSP, Hsp90, HSPA14, HSPB6, HSPB7, HSPB8, Ifn, IL1, IL32, IL12 (complex), IL12 (family), IL18RAP, IL1F6, IL1F8, IL1F9, Interferon alpha, Mmp, NELF, NFkB (complex), NPM3, ODF1, PIBF1, RNA polymerase II, SQSTM1, SUGT1, TAF10, WDR34, XAB2	17	14	Dermatological Diseases and Conditions, Genetic Disorder, Connective Tissue Disorders
Actin, ACTR8, ALDH9A1, APEX2, ARFIP1, ATL3, C12ORF45, CDC14B, CDCA7L, DCTD, DEPDC1, ELF4, KIAA1468, KIF4A, MIR124, MIR124-1 (includes EG:406907), MTMR6, NCAPD2, NCAPG (includes EG:64151), NCAPH, NECAP2, NEK6, OSBPL8, PFDN6, PTTG1IP, RDH, RDH10, RDH11, RDH13, RUVBL2, S100A11, SECISBP2, SEPW1, SURF4, TMEM109	17	14	DNA Replication, Recombination, and Repair, Cell Cycle, Cellular Assembly and Organization
12-hydroxyeicosatetraenoic acid, ADAM17, APBA3, C11ORF70, C4ORF49, CAMLG, CENPN, CNTF, EGFR, Egfr-Erbb2, FHOD1, G-protein beta, GRB2, Il8r, IRS1, MAP2K5, MIR122 (includes EG:406906), MRPL36, MYCN, NDN, NUBP1, NUMBL, ONECUT1, P110, PPP1R12C, PTGER1, RIN2, RNF122, RPS5, RPS15, RRAS, TAOK3, TMEM55B, UBAP2, ZKSCAN3	17	14	Cell Cycle, Connective Tissue Development and Function, Cancer
AIRE, ARMC1, BCORL1, DDX23, EFTUD2, FAM178B, GNL3, HIST1H2AC, KBTBD8, MIR138-1 (includes EG:406929), MIR138-2 (includes EG:406930), MIR183 (includes EG:406959), MIR221 (includes EG:407006), MYBBP1A, MYC, NCAPD3, NCAPG2, NEIL2, NFYB, NOP56, ODF2, PCNA, PNPT1, PRPF6, PRPF8, RNMTL1, SART1, SKIV2L, SMNDC1, SNRNP200, STX16, TSHZ1, TSR2, USP39, ZNF598	17	14	RNA Post-Transcriptional Modification, Cellular Development, Cellular Growth and Proliferation
ANXA1, C7ORF50, Ca2+, CALCA, CCDC86, DLST, EPO, FARSA, FAU, FOXP3, IL2, ITGA4, Laminin, METAP2, MRPS17, MTF2, PDCD2L, PINX1, POLR1A, POLR1B, POLR1C, POLR3K, PRNP, PTHLH, PTRH1, S100A11, SCNM1, sodium chloride, TBL3, TNFRSF11A, TNFSF9, TRPM4, USP11, USP25, WNT5A	17	14	Carbohydrate Metabolism, Small Molecule Biochemistry, Cell Morphology
ACIN1, ALDOA, AP2B1, ARRB1, C1ORF35, CHD1 (includes EG:1105), CLPB, DDX39, DPP3, FAM46C (includes EG:54855), GBAS, MPND (includes EG:84954), NSF, OSBPL10, PCBP3, PGAM5, RBM17, RNPS1, SAP18, SF3A2, SF3B2, SNAP29, SNRNP70, SNRPC, SRRM2, SS18L1, STX7, STX8, TIMM8A, USP30, VAMP7, VTI1A, VTI1B, WRNIP1, ZC3H18	15	13	RNA Post-Transcriptional Modification, Cellular Assembly and Organization, Cellular Compromise
AEN, C17ORF61, C8ORF30A, CDC34 (includes EG:997), CHFR, COX6C, DDX58, EXOSC9, FAM176A, FDPS, heparan sulfate, hydrogen peroxide, KIAA0776, MDM4, PSMD2, PTN, RFWD3, RLIM, RNF11, RNF181, TRAF7, TRIM25, TRIM26, TRIM31, TRIT1, UBA6, UBE2D1, UBE2D2, UBE2D3 (includes EG:7323), UBE2N, UBE2V1, UBE2V2, UBE4A, UBLCP1, UHRF2	15	13	Post-Translational Modification, Protein Degradation, Protein Synthesis
ATAD2, BDKRB1, C14ORF156, C21ORF122, CNR2, CXCR7, CYSLTR2, ESR1, FOS, Gpcr, GPR50, GPR172A, HESX1, IL9, LPAR1, LPAR2, LPAR3, MIR214 (includes EG:406996), MKL1, MKL2, NADSYN1, NR2C2, NR2C2AP, P2RY1, P2RY6, P2RY11, PISD, PTGER1, PTGER4, RPRML, RRP12, SMARCA4, SRF, STAT3, ZNF467	15	13	Tissue Morphology, Organismal Injury and Abnormalities, Gene Expression
ABCF3, ANGEL2, AP2A1, BARD1, C19ORF44, CP110, DNAJA2, FAM158A, GBF1, GPATCH3, HIST1H4K (includes EG:8362), HNF4A, KBTBD7, KIAA1967, LENG1, LLGL1, MRPL49, PI4KB, PJA2, PNMA1, POLRMT, RAP2C, RBM26, SPATA5L1 (includes EG:79029), TOE1, TP53, TRMT6, TRMT61A, TTC35, ZNF304	14	12	Infection Mechanism, Gene Expression, Cell Cycle
ATL1, C6ORF130, CCT5, CLK2, G protein alphai, G-protein beta, G-protein gamma, GNG2, GNG3, GNG4, GNG10, GNG13, GSN, HAVCR2, ITK, LGALS9, MUC1, NCF2, P2RY2, PNO1, PPP4R1, PPP4R2, PTGER4, RALGAPB, RTN4, SLC35A5, SMEK1, SMEK2, SPRR2A, SRC, SRM, SRPX, TGFB1, TNFRSF11A, UBL5	14	12	Inflammatory Response, Cellular Movement, Connective Tissue Development and Function
3-alpha,17-beta-androstanediol, 3-beta,17-beta-androstanediol, adenosine-tetraphosphatase, ATP5A1, ATP5B, ATP5C1, ATP5D, ATP5F1, ATP5G1, ATP5H (includes EG:10476), ATP5I, ATP5J, ATP5J2, ATP5L, ATP5S, ATP6V1H, beta-estradiol, CALCA, CGA, Creb, ERF, GTF2B, H+-transporting two-sector ATPase, Hat, HSD17B6, MAOA, MED21, MIR125B1, PARK2, PPP1R3F, PSENEN, RNU1-4, SYT11, ZNF91	12	11	Energy Production, Nucleic Acid Metabolism, Small Molecule Biochemistry

Molecules in Network	Score	Focus molecules	Top functions
MRPL10, USP22	2	1	Post-Translational Modification, Dermatological Diseases and Conditions, Infectious Disease

Supplementary Table S7: The list of 300 differentially expressed genes after overexpression of hsa-miR-195 in MDA MB-231 cells

GENE	FOL D CHANGE	GENE	FOL D CHANGE	GENE	FOL D CHANGE	GENE	FOL D CHANGE
OASL	17.06894	SGMS2	3.700018	PLEKHA4	2.439895	SNHG1	1.936489
HIST2H2BE	16.47681	NOV	3.640053	ZNF433	2.439837	ZNF614	1.913637
IFIT2	15.15934	KLF4	3.590595	LOC728014	2.371492	SNHG12	1.883175
TNFAIP3	14.58476	DDIT3	3.537082	ZNF165	2.365814	DCP1A	1.882825
GKN2	13.09462	PPP1R3C	3.497304	SDF2L1	2.320492	UTX	1.881167
EPSTI1	10.86279	HERC5	3.330319	BAMBI	2.318819	ZCCHC3	1.859662
ZNF442	9.638604	GADD45A	3.225136	HOXD1	2.315685	RP5-1022P6.2	1.85787
IFIT3	9.007547	LOC728473	3.189068	NRAS	2.295343	LOC644132	1.852055
EGR2	7.842551	TRAF1	3.132812	CRELD2	2.292652	FSTL3	1.844205
IFIH1	7.586282	SAMD9	3.104155	ZNF20	2.285483	ABTB2	1.835654
ISG20	7.227149	CPEB3	3.074315	NFKB2	2.283129	C2orf49	1.831587
ZNF844	6.877119	KIAA1199	3.050018	TINAGL1	2.281333	UFM1	1.831426
EGR1	6.876925	IGFBP6	3.020361	MXD1	2.276155	RNF103	1.830244
TXNIP	6.464297	RHEBL1	3.002876	CITED2	2.275989	C9orf6	1.823248
C7orf53	6.33289	TMEM49	2.99689	ZFP82	2.26644	IRF9	1.823126
RARRES3	5.901725	ARL5B	2.949305	LOC100130557	2.245823	UGCG	1.807145
PLCG2	5.874659	PPP1R15A	2.939112	SELM	2.235516	SPAG9	1.806158
IFI44	5.74208	HERPUD1	2.924858	NFKBIE	2.221349	MEIS3P1	1.80142
ZC3HAV1	5.49471	RELB	2.81942	NFKBIA	2.199819	ADAMTS1	1.799562
GBP4	5.478608	IFI6	2.817659	ZNF211	2.192035	ZNF317	1.79684
AURKAPS1	5.457495	CD55	2.792112	TIPARP	2.172029	CRISPLD2	1.794408
FLJ22639	5.343414	MAFF	2.789421	CCNL1	2.155736	TUBB2A	1.783384
ID2	5.320987	DNAJB9	2.780508	EVI2B	2.13973	SFRS17A	1.780933
MT1F	5.313781	OTUD1	2.751975	ZNF35	2.128735	ZBTB43	1.779689
PTGS2	5.296492	CEBPD	2.751596	PLK2	2.121539	TAF1D	1.77116
ISG15	5.190974	C5orf41	2.742389	USPL1	2.076638	LOC653506	1.770054
FAM46A	5.00128	CCL5	2.698931	SQSTM1	2.058003	NCOA7	1.769459
PMAIP1	4.995643	SIPA1L2	2.680575	MYLIP	2.052192	SPANXB2	1.768877
DNAJC27	4.929485	CCRN4L	2.670748	CBLB	2.037692	ZNF296	1.765277
MAL2	4.63144	LOXL4	2.622707	RAB30	2.036317	ARID5B	1.761003
CLIC3	4.620813	SDC4	2.607125	BIRC3	2.026914	FLJ38717	1.755854
HIST1H2BE	4.261324	DUSP10	2.571684	SLC30A1	2.025615	TSC22D1	1.754877
IL8	4.167474	CENTA1	2.567407	FZD4	2.007027	VEGFC	1.754647
LOC400750	3.982144	PTGER4	2.554784	KIAA1370	1.985903	ZNFX1	1.733294
HERC6	3.971393	ZNF26	2.492114	JUN	1.978861		1.729764
JUNB	3.938018	ZNF34	2.457071	NFIL3	1.959953	IER2	1.729639
SLC25A24	3.870423	LOC730020	2.453708	GADD45B	1.948742	LOC400027	1.725512
CYP1B1	3.836099	ZNF140	2.449402	TARDBP	1.94607	FAM179B	1.724479

GENE	FOL D CHANGE	GENE	FOL D CHANGE	GENE	FOL D CHANGE	GENE	FOL D CHANGE
ZFAND2A	1.717806	ADM	1.672243	U2AF1L2	1.646148	ATF4	1.502976
SCYL2	1.713385	USP36	1.671429	RSRC2	1.640113	POFUT2	1.497713
RABGGTB	1.710804	TBCC	1.655036	CCDC49	1.637061	STK19	1.497579
ZNF17	1.709293	MOAP1	1.647446	TSPAN8	1.634878	WBP4	1.496859
PDRG1	1.704679	U2AF1L2	1.646148	MYC	1.633516	CIR1	1.484091
C1orf52	1.704209	RSRC2	1.640113	RFX1	1.626561	ARMET	1.483472
C20orf111	1.697283	CCDC49	1.637061	C10orf137	1.624372	BRD2	1.476724
LOC388796	1.69455	TSPAN8	1.634878	PTBP2	1.622112	DNAJB11	1.469135
TDRD7	1.693787	MYC	1.633516	CHMP1B	1.620053	NFE2L2	1.467157
FGFR1OP2	1.693712	RFX1	1.626561	RND3	1.611452	CALR	1.465663
HYOU1	1.687914	C10orf137	1.624372	RNF44	1.607222	COQ10B	1.455646
EAF1	1.686408	PTBP2	1.622112	CLK1	1.597759	RYBP	1.450223
TNIP1	1.682705	CHMP1B	1.620053	TMEM170A	1.593665	UBAP1	1.449914
C6orf111	1.678955	RND3	1.611452	ZNF721	1.592432	MAT2A	1.446107
ADNP2	1.674116	RNF44	1.607222	SFRS18	1.588286	NXF1	1.444505
ADM	1.672243	CLK1	1.597759	FAM53C	1.58568	ATP6V0A1	1.444491
USP36	1.671429	TMEM170A	1.593665	TIGA1	1.574824	SERTAD1	1.441112
TBCC	1.655036	ZNF721	1.592432	IFITM3	1.57379	BTG1	1.441075
MOAP1	1.647446	SFRS18	1.588286	MTF1	1.573158	IER5	1.440759
U2AF1L2	1.646148	FAM53C	1.58568	TUFT1	1.571844	C19orf10	1.425351
RSRC2	1.640113	TIGA1	1.574824	C3orf19	1.566994	BCL2L2	1.423364
CCDC49	1.637061	IFITM3	1.57379	SNIP1	1.566522	SPRY2	1.414353
TSPAN8	1.634878	MTF1	1.573158	KLF5	1.563255	PPTC7	1.412823
MYC	1.633516	TUFT1	1.571844	TBC1D15	1.562179	DERL2	1.405918
RFX1	1.626561	C3orf19	1.566994	LOC143666	1.559991	SEC11C	1.401056
C10orf137	1.624372	SNIP1	1.566522	PNPLA2	1.553494	NFKB1	1.399158
PTBP2	1.622112	KLF5	1.563255	MBIP	1.553335	BCL2L13	1.395131
CHMP1B	1.620053	TBC1D15	1.562179	MAP1LC3B	1.552862	SAT2	1.387294
ZFAND2A	1.717806	LOC143666	1.559991	PCGF1	1.549372	CHMP1A	1.379048
SCYL2	1.713385	PNPLA2	1.553494	LOC100132715	1.546334	CCND3	1.367315
RABGGTB	1.710804	MBIP	1.553335	ZNF207	1.543732	KPNA4	1.364454
ZNF17	1.709293	MAP1LC3B	1.552862	MIDN	1.538633	C12orf47	1.364026
PDRG1	1.704679	PCGF1	1.549372	ATG12	1.537057	EIF1	1.348476
C1orf52	1.704209	LOC100132715	1.546334	ELF1	1.530592	AHR	1.027235
C20orf111	1.697283	ZNF207	1.543732	CABLES1	1.526789	TUBA1A	0.878808
LOC388796	1.69455	MIDN	1.538633	KIF5B	1.525714	CMTM7	0.74677
GENE	FOL D CHANGE	GENE	FOL D CHANGE	GENE	FOL D CHANGE	GENE	FOL D CHANGE

TDRD7	1.693787	ATG12	1.537057	MAP2K1IP 1	1.525342	LOC730744	0.738232
FGFR1OP2	1.693712	ELF1	1.530592	SLU7	1.523212	LOC402175	0.735079
HYOU1	1.687914	CABLES1	1.526789	KRT8	1.51789	HNRPUL1	0.726829
EAF1	1.686408	ADM	1.672243	RRAGC	1.512836	UBE4B	0.725045
TNIP1	1.682705	USP36	1.671429	HNRPDL	1.508895	FADD	0.723624
C6orf111	1.678955	TBCC	1.655036	IFITM2	1.506512	RNASEH2A	0.72083
ADNP2	1.674116	MOAP1	1.647446	CYR61	1.503113	LOC641814	0.705275
LOC653186 0.70269	0.70269	LOC64491 4	0.675202	CENTG2	0.657989	FAM171A1	0.635709
UBE2C	0.702552	LOC10012 7888	0.673982	COL13A1	0.651101	SLCO4A1	0.635489
LOC440926	0.699038	CYP2R1	0.667403	LOC641849	0.648189	PPPDE1	0.634665
NCAPD2	0.696543	FANCG	0.66346	TGOLN2	0.644694	PATL1	0.630256
LOC390466	0.693594	IPO9	0.663191	C18orf55	0.642106	SPC24	0.626878
HIRIP3	0.678618	MRPS16	0.660576	CAMK2N1	0.640622	KRI1	0.617245
LOC729964	0.675395	E2F2	0.65874	HNRNPA0	0.639678	TCFL5	0.610155
LOC727761	0.609679	KANK2	0.555448	UCN2	0.530354	KIF15	0.57749
C5orf21	0.607568	TBL1X	0.550564	NT5DC2	0.518712	VEGFA	0.539428
LOC92755	0.607203	FLJ12949	0.547968	NPLOC4	0.468267	TCEB3	0.270787

Supplementary Table S8: The list of 219 differentially expressed genes after depletion of hsa-miR-195 in MDA MB-231 cells

GENE	FOL D CHANGE	GENE	FOL D CHANGE	GENE	FOL D CHANGE	GENE	FOL D CHANGE
MT1F	12.92171	LOC283953	3.782871	CST2	3.319776	DPYSL3	3.171952
GDF15	12.34118	KLHL24	3.702341	ASNS	3.287984	PLEKHF1	3.162213
HMOX1	8.011292	WIPI1	3.663805	PDIA4	3.224833	MVD	3.148995
MUC5AC	7.750373	CLIC3	3.654506	DDIT4	3.219646	C17orf91	3.147308
S100P	7.730428	FICD	3.565174	ZBTB20	3.209615	ABHD4	3.134516
PARM1	7.121587		3.543995	LOC158160	3.191313	NDRG1	3.014372
AGR2	6.929166	TMEM150A	3.440866	DPYSL3	3.171952	EDEM1	2.997954
NUPR1	6.866367	PCSK9	3.374442	PLEKHF1	3.162213	SEC24D	2.989821
LOC400750	6.605076	ITGA10	3.349686	MVD	3.148995	LOC642567	2.988706
PTGS2	6.601668	RARRES3	3.330806	C17orf91	3.147308	CBLB	2.983402
SEL1L3	6.448566	CST2	3.319776	ABHD4	3.134516	GLT25D2	2.95367
FTH1	6.205678	ASNS	3.287984	NDRG1	3.014372	FTHL2	2.938135
C17orf28	6.1478	PDIA4	3.224833	EDEM1	2.997954	LZTFL1	2.900801
HSPA5	6.145937	DDIT4	3.219646	SEC24D	2.989821	TP53INP2	2.889429
DDIT3	6.017756	ZBTB20	3.209615	LOC642567	2.988706	IGDCC4	2.880479
TRIB3	5.860122	LOC158160	3.191313	CBLB	2.983402	GLRX	2.870043
PIK3IP1	5.762268	DPYSL3	3.171952	GLT25D2	2.95367	WARS	2.849307
TXNIP	5.591399	PLEKHF1	3.162213	FTHL2	2.938135	UGDH	2.849168
DNAJB9	5.525446	MVD	3.148995	LZTFL1	2.900801	KIAA0182	2.845813
HMGCS1	5.451573	C17orf91	3.147308	TP53INP2	2.889429	LBA1	2.816353
MAL2	5.225329	ABHD4	3.134516	IGDCC4	2.880479	MIR1974	2.813848
HERPUD1	5.136469	NDRG1	3.014372	GLRX	2.870043		2.801774
ISG20	4.993204	EDEM1	2.997954	WARS	2.849307	GABARAPL1	2.795643
IL24	4.836435	SEC24D	2.989821	UGDH	2.849168	DHCR7	2.791779
LOC651309	4.762677	LOC642567	2.988706	KIAA0182	2.845813		2.776056
OASL	4.656957	CBLB	2.983402	LBA1	2.816353	SQSTM1	2.764781
SESN2	4.537008	GLT25D2	2.95367	MIR1974	2.813848	HYOU1	2.755111
CYP1B1	4.530764	FTHL2	2.938135		2.801774	FTHL11	2.747572
TP53INP1	4.502775	LOC283953	3.782871	GABARAPL1	2.795643	YPEL3	2.731676
STOX2	4.391771	KLHL24	3.702341	CST2	3.319776	SC5DL	2.708963
SDF2L1	4.351683	WIPI1	3.663805	ASNS	3.287984	LOC729009	2.701955
GENE	FOL D	GENE	FOL D	GENE	FOL D	GENE	FOL D

	CHANGE		CHANGE		CHANGE		CHANGE
TMEM50B	4.322087	CLIC3	3.654506	PDIA4	3.224833	IFI44	2.700555
CRELD2	4.313261	FICD	3.565174	DDIT4	3.219646	BTN3A3	3.848782
IL6	4.131138		3.543995	ZBTB20	3.209615	CTH	3.840209
SPINK4	4.093725	TMEM150A	3.440866	LOC158160	3.191313	ITGA10	3.349686
ANG	3.858606	PCSK9	3.374442	GPT2	2.259324	RARRES3	3.330806
NPEPL1	2.686811	CST4	2.666683	ANKRD29	2.255791	CALR	1.994345
CYP4V2	2.681988	PLD1	2.66507	BHLHB2	2.254975	GFPT1	1.981992
FDFT1	2.681575	AKR1C3	2.650522	ISG15	2.237997	FTHL16	1.977865
CST4	2.666683		2.647828	NOV	2.236305	TICAM2	1.973222
PLD1	2.66507	C5orf41	2.632938		2.229196	FNDC3B	1.964631
AKR1C3	2.650522	KYNU	2.615341	TRIM2	2.218492	ENPP1	1.940911
	2.647828	ARMET	2.61024	SNTB1	2.217562	YPEL5	1.932436
C5orf41	2.632938	FAIM3	2.610073	MXD4	2.214417	CEBPB	1.916925
KYNU	2.615341	INSIG1	2.602511	SLC3A2	2.209719	DNAH2	1.905113
ARMET	2.61024	CDRT4	2.59641	TOM1	2.208385	ALDH3A2	1.897843
FAIM3	2.610073		2.585514	CSF2	2.203935	DNASE2	1.881188
INSIG1	2.602511	LOC284988	2.578735	LOC100134294	2.20161	ST3GAL6	1.880142
CDRT4	2.59641	SYVN1	2.537388	SQLE	2.199471	C20orf127	1.879554
	2.585514	RASD1	2.525786	FTHL3	2.194639	NPC1	1.87247
LOC284988	2.578735	CYP51A1	2.524228	SIK1	2.177892	MAP1LC3B	1.870552
SYVN1	2.537388		2.51016	MEF2D	2.167861	DERL2	1.867962
RASD1	2.525786	NUCB2	2.508457	MVP	2.164221	TMEM41B	1.865482
CYP51A1	2.524228	RHOB	2.501659	EDEM2	2.157266	CANX	1.861614
	2.51016	NR4A2	2.472255	C4orf34	2.143418	GARS	1.858383
NUCB2	2.508457	NQO1	2.441649	SLC9A3R1	2.13799	PHGDH	1.855167
RHOB	2.501659	ANKRD33	2.427613	RBCK1	2.136853	MT1G	1.8268
NR4A2	2.472255	LOC390940	2.398996	HERC6	2.126279	NEU1	1.822984
NQO1	2.441649	ME1	2.395729	BEX2	2.110272	FADS1	1.819838
ANKRD33	2.427613	HBP1	2.38926	FTHL8	2.084453	FAM46A	1.81892
LOC390940	2.398996	GBP2	2.366766	TRA1P2	2.079019	DNAJB11	1.818558
ME1	2.395729	C7orf54	2.356919	ANGPTL2	2.074981	NSDHL	1.814089
HBP1	2.38926	IDI1	2.344755	SLC2A3	2.071603	PTGES	1.810826
GBP2	2.366766	KLF9	2.340772	STX3	2.057944	OSBP	1.807698
C7orf54	2.356919	IFIT2	2.325264	MT1X	2.050466	MFGE8	1.792728
IDI1	2.344755	XBP1	2.312835	HMGCR	2.046453	ARHGEF3	1.792677
KLF9	2.340772	TMEM154	2.311618	GPX3	2.040075	CREB3L2	1.791394
IFIT2	2.325264	LOC390530	2.308309	CLIC6	2.034932	BRI3P1	1.7851
XBP1	2.312835	CDK2AP2	2.303611	CARS	2.025904	TNIP1	1.769195
GENE	FOL D	GENE	FOL D	GENE	FOL D	GENE	FOL D

	CHANGE		CHANGE		CHANGE		CHANGE
TMEM154	2.311618	TFF3	2.301527	LGMN	2.025268	MSTO1	1.749158
CDK2AP2	2.303611	HSP90B1	2.294493	LOC88523	2.003911	TM7SF2	1.745965
TFF3	2.301527	OPTN	2.288713	RHBDF1	1.742093	DKK1	0.642096
NPEPL1	2.686811	GMPPA	2.285267	BRI3	1.728167	TMEM137	0.57071
CYP4V2	2.681988	FTHL12	2.27762	BLVRA	1.674488	LOC100128337	0.563919
FDFT1	2.681575	ZC3H12C	2.27683	ATP6V0A1	1.663229	NCAPG2	0.535504
LOC390530	2.308309	KIAA1539	2.300405	WFS1	1.634925	KIF15	0.517118
LOC100133372	0.51672	RPL29	0.493789	SLC39A10	0.460624		
AOX1	0.494994	RPS7	0.474291	H2AFX	0.435937		
